# YAP and TAZ protect against white adipocyte cell death during obesity

**DOI:** 10.1038/s41467-020-19229-3

**Published:** 2020-10-28

**Authors:** Lei Wang, ShengPeng Wang, Yue Shi, Rui Li, Stefan Günther, Yu Ting Ong, Michael Potente, Zuyi Yuan, Enqi Liu, Stefan Offermanns

**Affiliations:** 1 Department of Pharmacology, Max Planck Institute for Heart and Lung Research, Bad Nauheim, 61231 Germany; 2 Cardiovascular Research Center, School of Basic Medical Sciences, Xi’an Jiaotong University Health Science Center, Yanta District, Xi’an, China; 3 Bioinformatics and Deep Sequencing Platform, Max Planck Institute for Heart and Lung Research, Bad Nauheim, 61231 Germany; 4 Angiogenesis and Metabolism Laboratory, Max Planck Institute for Heart and Lung Research, Bad Nauheim, 61231 Germany; 5 Department of Cardiology, First Affiliated Hospital of Xi’an Jiaotong University, Xi’an, China; 6 Laboratory Animal Center, Xi’an Jiaotong University Health Science Center Xi’an Jiaotong University, Xi’an, China; 7 Center for Molecular Medicine, Medical Faculty, Goethe University, Frankfurt am Main, 60590 Germany

**Keywords:** Fat metabolism, Obesity

## Abstract

The expansion of the white adipose tissue (WAT) in obesity goes along with increased mechanical, metabolic and inflammatory stress. How adipocytes resist this stress is still poorly understood. Both in human and mouse adipocytes, the transcriptional co-activators YAP/TAZ and YAP/TAZ target genes become activated during obesity. When fed a high-fat diet (HFD), mice lacking YAP/TAZ in white adipocytes develop severe lipodystrophy with adipocyte cell death. The pro-apoptotic factor BIM, which is downregulated in adipocytes of obese mice and humans, is strongly upregulated in YAP/TAZ-deficient adipocytes under HFD, and suppression of BIM expression reduces adipocyte apoptosis. In differentiated adipocytes, TNFα and IL-1β promote YAP/TAZ nuclear translocation via activation of RhoA-mediated actomyosin contractility and increase YAP/TAZ-mediated transcriptional regulation by activation of c-Jun N-terminal kinase (JNK) and AP-1. Our data indicate that the YAP/TAZ signaling pathway may be a target to control adipocyte cell death and compensatory adipogenesis during obesity.

## Abstract (web summary)

The expansion of the white adipose tissue during obesity is accompanied by increased cellular stress, but factors that protect adipocytes from cell death are not well known. Here the authors report that the transcriptional co-activators YAP and TAZ are activated in adipocytes during obesity, which increases adipocyte survival through the proapoptotic factor BIM.

## Introduction

Obesity is a serious health problem as it is a strong risk factor for the development of type-2 diabetes, cardiovascular diseases, and cancer^1–6^. In obesity, the adipose tissue undergoes dynamic remodeling including an increase in size (hypertrophy) and number of adipocytes (hyperplasia), an infiltration by immune cells, and the development of tissue fibrosis^7–12^. In the obese adipose tissue, adipocytes are exposed to different forms of stress. This includes hypoxia, oxidative stress, metabolic stress, mechanical stress from hypertrophy, and inflammatory stress due to infiltration of the adipose tissue by immune cells and increased levels of cytokines^13–19^. Different forms of cellular stress can activate extrinsic and intrinsic pathways of cell death resulting in adipocyte apoptosis^20–23^. There are also anti-apoptotic, pro-survival pathways activated in adipocytes of the obese adipose tissue, which protect adipocytes against cell death. This is indicated by various mouse models with defects in pro-survival pathways, which show strongly increased adipocyte death during obesity^24–26^. However, the mechanisms protecting adipocytes from uncontrolled cell death during obesity are still poorly understood.

YAP and its paralog TAZ are transcriptional cofactors, which regulate various cell functions including cell proliferation, differentiation, and cell survival by integrating different cellular signaling pathways^27–30^. Their nuclear translocation and activity are controlled by several upstream signaling pathways. Activation of the Hippo pathway results in phosphorylation of YAP/TAZ by LATS kinase, leading to the cytoplasmic retention and degradation of YAP/TAZ^30–32^. In addition, G-protein-mediated signaling pathways involving G_12_/G_13_ or G_q_/G_11_^33,34^, as well as extracellular matrix stiffness and cytoskeletal tension, can promote nuclear translocation and activation of YAP/TAZ^28,35–37^. Nuclear YAP/TAZ can co-activate or co-repress numerous genes involved in cell proliferation, differentiation, and survival by interacting with different transcription factors of which the TEAD (TEA domain) family transcription factors are the best characterized^32,38–40^. Given that YAP and TAZ play vital functions in cell survival, we hypothesized that YAP and TAZ are crucial for adipocyte homeostasis.

Here we show that adipocyte YAP and TAZ are activated during obesity and induce upregulation of anti-apoptotic and downregulation of pro-apoptotic factors such as BIM (Bcl-2 interacting mediator of cell death), and thereby promote adipocyte survival in diet-induced obesity.

## Results

### Obesity promotes activation of YAP and TAZ in human and mouse white adipocytes

**Fig. 1:**
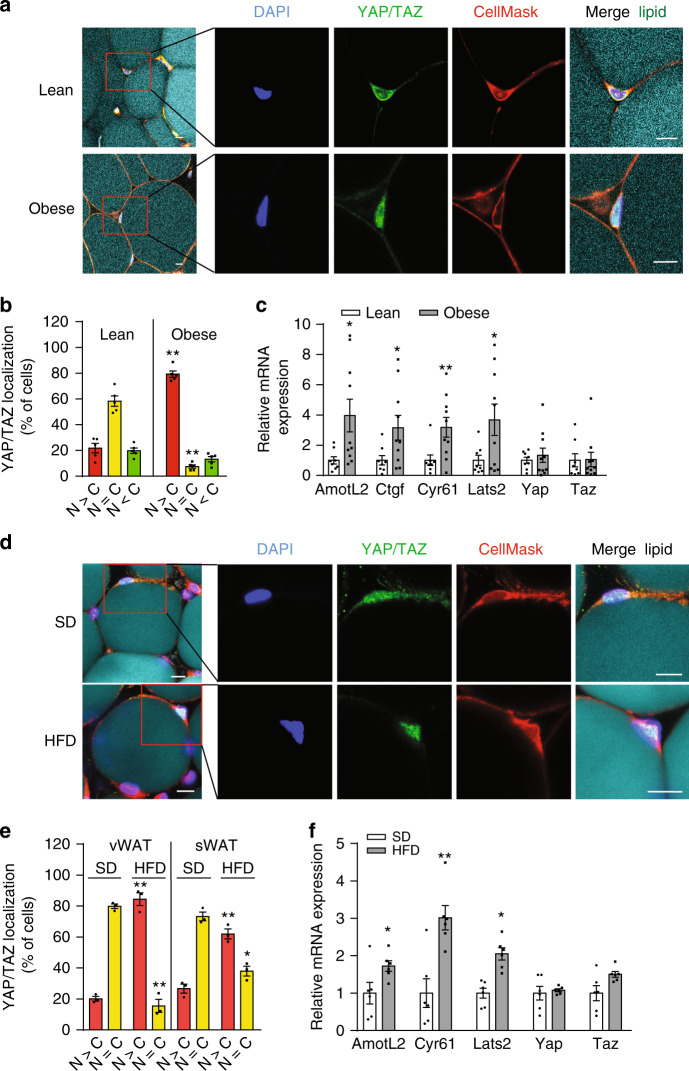
Obesity promotes YAP/TAZ activation in white adipocytes. **a**, **d** vWAT from lean (body mass index < 25) and obese subjects (body mass index > 30) (**a**) or from mice fed HFD for 4 weeks and mice fed standard diet (SD) (**d**) was stained with anti-YAP/TAZ antibody, DAPI, CellMask to visualize adipocyte plasma membranes and LipidTOX (lipid). Scale bars: 10 µm. **b**, **e** Quantification of YAP/TAZ localization to the nucleus (N) or cytosol (C) in visceral white adipocyte of human lean (*n* = 5), and obese subjects (*n* = 5) (**b**) or in the visceral (vWAT) and subcutaneous white adipose tissue (sWAT) from standard diet (SD)-fed (*n* = 3) and HFD-fed mice (*n* = 3) (**e**). **c**, **f** Expression of YAP/TAZ target genes, and of Yap and Taz in white adipocyte isolated from human visceral adipocytes of lean (*n* = 8) and obese subjects (*n* = 10) (**c**) or from the vWAT of mice fed HFD (*n* = 6) or standard diet (SD) (*n* = 6) for 4 weeks (**f**). Data are presented as the mean ± SEM; *p ≤ 0.05 and **p ≤ 0.01 (compared to lean humans or standard diet (SD)-fed mice in **b**, **e**) (unpaired Student’s *t*-test). Source data are provided as a Source Data file.

To test whether YAP and TAZ activity in white adipocytes is affected during obesity, we examined the subcellular localization of YAP and TAZ, as well as YAP/TAZ target gene expression in white adipocytes of humans and mice. In human visceral adipocytes from lean subjects (body mass index < 25), YAP and TAZ were localized equally in the cytosol and nucleus, whereas YAP/TAZ were predominantly detected in the nuclei of human visceral adipocytes from obese subjects (body mass index > 30) (Fig. 1a, b). Quantitative reverse transcriptase PCR (RT-PCR) showed that expression of YAP/TAZ target genes, such as *Cyr61*, *AmotL2*, and *Lats2*, was upregulated in adipocytes of obese subjects (Fig. 1c). Similarly, in adipose tissue from mice fed a standard diet (SD), YAP and TAZ were localized both in the cytosol and nucleus (Fig. 1d, e), whereas 4 weeks of high-fat diet (HFD) feeding resulted in a strong increase in nuclear localization of YAP/TAZ (Fig. 1d, e), both in the epididymal (visceral) and the inguinal (subcutaneous) white adipose tissue (WAT). Quantitative RT-PCR showed increased expression of YAP/TAZ target genes in adipocytes from HFD-fed mice (Fig. 1f). These data indicate that obesity in humans and mice goes along with increased activation of YAP and TAZ in white adipocytes.

### Adipocyte-specific YAP and TAZ deficiency leads to increased adipocyte death during obesity

**Fig. 2:**
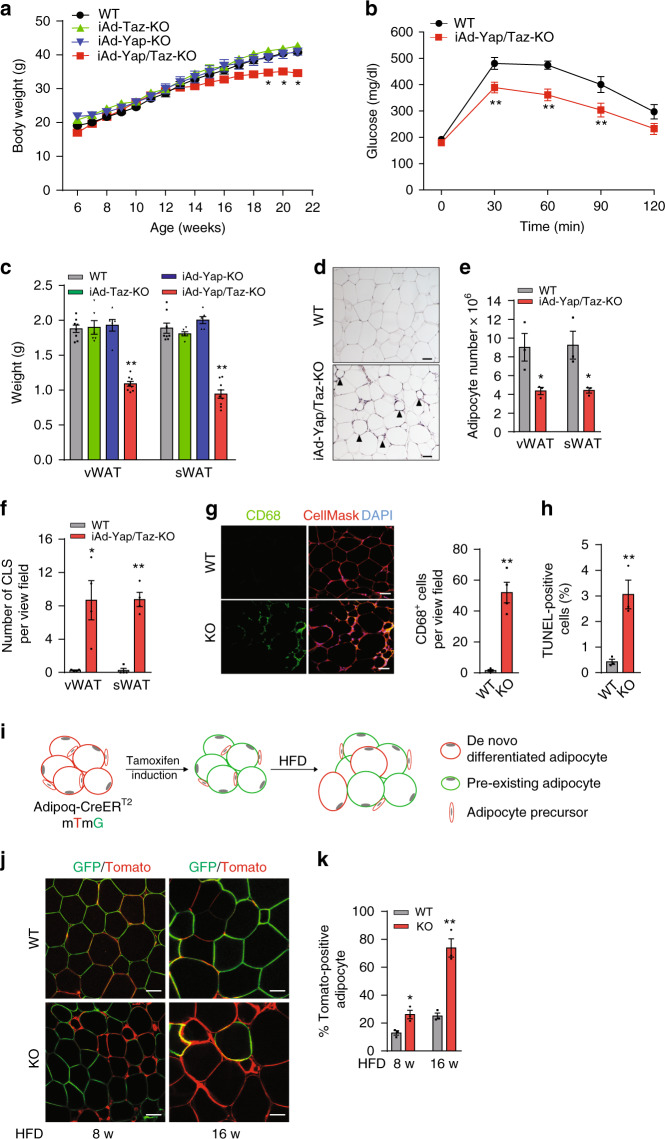
Induction of Yap/Taz loss in mature white adipocyte leads to adipose death and increased adipogenesis during HFD feeding. **a**–**c** Wild-type (WT, *n* = 8–9), iAd-Yap/Taz-KO (*n* = 9–11), iAd*-*Yap-KO (*n* = 6–7), and iAd*-*Taz-KO (*n* = 6) mice were fed HFD for 16 weeks. Shown are the body weight development (**a**), glucose tolerance (**b**), and weight of vWAT and sWAT (**c**) after 16 weeks of HFD. **d** H&E-stained epididymal vWAT sections from WT and iAd-Yap/Taz-KO mice after 16 weeks of HFD. Arrowheads point to crown-like structures. Scale bar: 50 µm. **e** Total adipocyte number in vWAT and sWAT from WT and iAd-Yap/Taz-KO mice fed HFD for 16 weeks (*n* = 3 mice in both groups; at least 10 sections were analyzed per animal). **f** Number of crown-like structures (CLS) in vWAT and sWAT from WT and iAd-Yap/Taz-KO mice fed HFD for 16 weeks (*n* = 4 mice per group; at least 10 view fields were analyzed per animal). **g** Sections of vWAT from WT and iAd-Yap/Taz-KO mice stained with anti-CD68 antibody (green), CellMask (red), and DAPI. Scale bar: 50 µm. The bar diagram shows the statistical evaluation of CD68-positive cells per view field (*n* = 4 mice per group; at least 10 sections per animal were analyzed). **h** TUNEL-positive cells in sections of vWAT from WT and iAd-Yap/Taz-KO mice (*n* = 3 mice per group; at least 10 sections per animal were analyzed). **i** Experimental design of analysis of adipogenesis in vivo using Adipoq-CreER^T2^;mT/mG mice. **j**, **k** Representative images (**j**) and statistical analysis (**k**) of adipocyte tracing in Adipoq-CreER^T2^;mT/mG; Yap^flox/flox^;Taz^flox/flox^ mice (KO), and Adipoq-CreER^T2^;mT/mG mice (WT) after tamoxifen treatment and 8 and 16 weeks of HFD (*n* = 3 mice; at least 10 sections per animal were analyzed). Scale bars: 50 µm. Shown are mean values ± SEM; *p ≤ 0.05 and **p ≤ 0.01 (two-way ANOVA in **a**, **b** and one-way ANOVA in **c**, and unpaired Student’s *t*-test in **e**–**h**, **k**). Source data are provided as a Source Data file.

To examine the functions of YAP and TAZ in white adipocytes, we generated inducible adipocyte-specific YAP- and TAZ-deficient mice by crossing mice carrying floxed alleles of Yap and Taz (Yap^fl/fl^;Taz^fl/fl^) with the Adipoq-CreER^T2^ mouse line^41^. Adipocyte-specific, tamoxifen-inducible Yap/Taz-deficient mice (Adipoq-CreER^T2^; Yap^fl/fl^;Taz^fl/fl^, hereafter termed iAd*-*Yap/Taz-KO) showed strong recombination of both floxed alleles in white adipocytes, resulting in loss of YAP and TAZ proteins (Supplementary Fig. 1a). On SD, iAd*-*Yap/Taz-KO mice showed no difference in total body weight, glucose tolerance, or fat mass compared to wild-type littermates (Supplementary Fig. 1b–d). To test whether activation of YAP and TAZ during obesity affects adipocyte function, iAd-Yap/Taz-KO mice were fed a HFD. After 12 weeks of HFD feeding, iAd-Yap/Taz-KO mice gained significantly less weight compared to wild-type littermates (Fig. 2a). Fasting blood glucose levels were similar between both groups, but iAd-Yap/Taz-KO mice showed an improved glucose tolerance compared to wild-type littermates (Fig. 2b). The mass of the visceral WAT (vWAT) and subcutaneous WAT (sWAT) of iAd-Yap/Taz-KO mice was markedly reduced compared to wild-type littermates (Fig. 2c), whereas body size and mass of other tissues including interscapular brown adipose tissue (BAT) was unchanged (Supplementary Fig. 2a, b). These changes were not observed in mice with induced adipocyte-specific loss of YAP or TAZ alone (Fig. 2a, c and Supplementary Fig. 2c), indicating that both transcriptional regulators have overlapping functions in adipocytes. Adipocyte morphology and average cell size were not changed in iAd-Yap/Taz-KO mice (Fig. 2d and Supplementary Fig. 2d, e), but the total number of adipocytes was strongly reduced in the visceral and sWAT (Fig. 2e). We also found an increased number of crown-like structures and of macrophages as well as an increased number of cells undergoing apoptosis as determined by TUNEL (terminal deoxynucleotidyl transferase dUTP nick end labeling) assay in the WAT of iAd-Yap/Taz-KO mice (Fig. 2d, f–h). These data indicate that loss of YAP and TAZ results in increased adipocyte death during obesity.

Despite very high recombination efficiency in adipocytes resulting in loss of YAP and TAZ shortly after tamoxifen treatment (Supplementary Fig. 1a), we noticed that, after 16 weeks of HFD, Yap/Taz expression was similar to wild-type mice (Supplementary Fig. 2f). To test whether the increased adipocyte cell death due to loss of YAP and TAZ leads to increased adipogenesis resulting in accumulation of newly formed wild-type adipocyte in iAd*-*Yap/Taz-KO mice, we performed adipocyte pulse-chase experiments^42^. We therefore crossed adipocyte-specific, tamoxifen-inducible Cre transgenic mice (Adipoq-CreER^T2^) with the mT/mG Cre reporter line, which switches from membrane-targeted Tomato expression to membrane-targeted enhanced green fluorescent protein (EGFP) expression upon Cre-mediated recombination (Fig. 2i)^43^. One week after tamoxifen injection, nearly all adipocytes showed green fluorescence (Supplementary Fig. 2g), and 8 weeks after induction of Cre activity and feeding with SD, 4–5% of the adipocytes in the vWAT of both wild-type and iAd-Yap/Taz-KO mice were Tomato-positive (Supplementary Fig. 2h). However, whereas in wild-type mice fed an HFD for 8 and 16 weeks, 13% and 25% of adipocytes were Tomato-positive (Fig. 2j, k), 26% and 74% of adipocytes showed red fluorescence in adipocyte-specific Yap/Taz knockouts (Fig. 2j, k). This indicates that they had formed from Tomato-positive adipocyte precursor cells after induction with tamoxifen and HFD feeding. Thus, loss of adipocyte YAP/TAZ in obese iAd*-*Yap/Taz-KO mice leads to increased adipocyte death and increased adipogenesis resulting in the replacement of the majority of YAP/TAZ-deficient adipocytes by wild-type adipocytes after a few months.

### Constitutive loss of adipocyte YAP and TAZ leads to adipocyte death and lipodystrophy under HFD

**Fig. 3:**
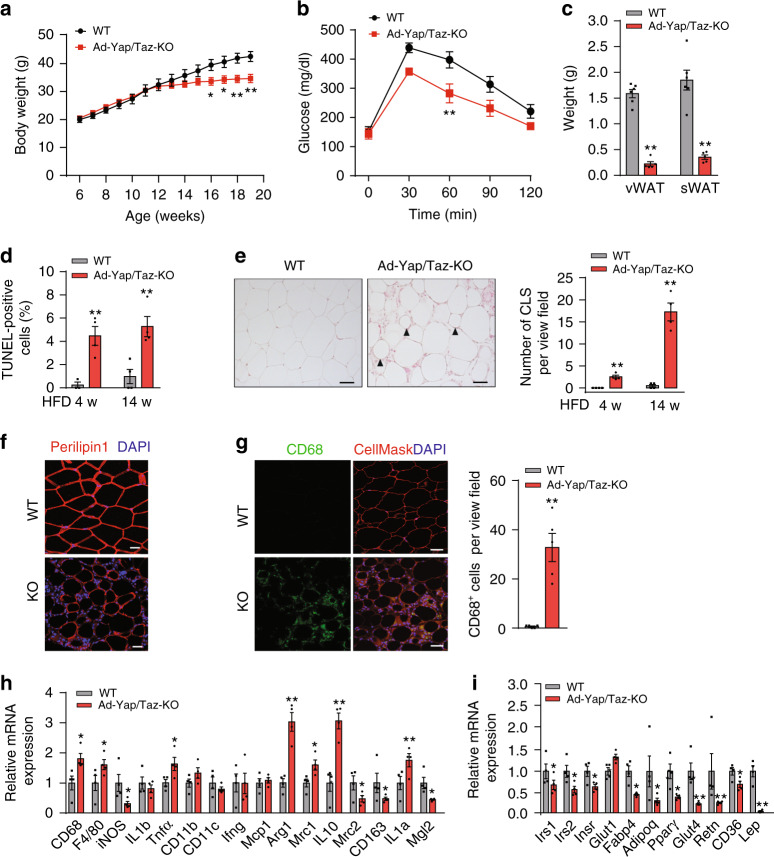
Ad-Yap/Taz-KO mice exhibit increased adipocyte cell death and lipodystrophy. **a**–**c** Wild-type (WT, *n* = 6) and Ad-Yap/Taz-KO mice (*n* = 5–8) were fed HFD for 14 weeks starting at 6 weeks of age. Shown are the development of body weight (**a**), glucose tolerance after 12 weeks of HFD (**b**), as well as weight of vWAT and sWAT (**c**). **d**, **e** Wild-type (WT, *n* = 3–4) and Ad-Yap/Taz-KO mice (*n* = 4) were fed HFD for 4 or 14 weeks (w). Shown are the quantification of TUNEL-positive cells in sections of vWAT from WT and Ad-Yap/Taz-KO mice (**d**), and H&E-stained epididymal vWAT sections (14 weeks) (**e**). Arrowheads in **e** indicate crown-like structures (CLS). Scale bar: 50 µm. The bar diagram (**e**) shows the number of CLS in vWAT from WT and Ad-Yap/Taz-KO mice (*n* = 4 mice in both groups; at least 10 sections were analyzed per animal). **f**, **g** Paraffin sections of vWAT from WT and Ad-Yap/Taz-KO mice stained with anti-Perilipin-1 (red) and DAPI (**f**) or with anti-CD68 antibody (green), CellMask (red), and DAPI (**g**). Scale bars: 50 µm. The bar diagram shows the statistical evaluation of CD68^+^ cells per view field (*n* = 5 mice per group; at least 10 sections were analyzed per animal). **h**, **i** Quantitative RT-PCR showing mRNA expression of M1 and M2 macrophage marker genes (**h**) and of adipocyte-specific marker genes (**i**) in vWAT from WT and Ad-Yap/Taz-KO mice fed HFD for 14 weeks (*n* = 4 mice per group). Data are presented as the mean ± SEM; *p ≤ 0.05 and **p ≤ 0.01 (two-way ANOVA in **a**, **b** and unpaired Student’s *t*-test in **c**–**e**, **g**–**i**). Source data are provided as a Source Data file.

To avoid replacement of Yap/Taz-deficient adipocytes by wild-type adipocytes in iAd-Yap/Taz-KO mice, we generated constitutive adipocyte-specific Yap/Taz-deficient mice by crossing Yap^fl/fl^;Taz^fl/fl^ mice with Adipoq*-*Cre mice^44^ (Adipoq-Cre;Yap^fl/fl^;Taz^fl/fl^, henceforth termed Ad-Yap/Taz-KO), which were born at the expected Mendelian ratio without any apparent abnormality. On SD, Ad*-*Yap/Taz-KO mice showed normal total body weight and glucose tolerance (Supplementary Fig. 3a, b). There was also no difference in fat tissue mass between Ad-Yap/Taz-KO mice and wild-type animals, but Ad*-*Yap/Taz-KO mice showed a slight increase in crown-like structures in their adipose tissue (Supplementary Fig. 3c, d). However, after 10 weeks of HFD feeding, the total body weight of Ad*-*Yap/Taz-KO mice started to be significantly lower than that of wild-type littermates (Fig. 3a). This was accompanied by an increased glucose tolerance and a massive reduction in the mass of both vWAT and sWAT in Ad-Yap/Taz-KO mice (Fig. 3b, c). Histological analysis of adipose tissue from HFD-fed Ad-Yap/Taz-KO mice showed increased numbers of TUNEL-positive adipocytes undergoing apoptosis as well as increased numbers of crown-like structures already after 4 weeks of HFD feeding (Fig. 3d, e). After 14 weeks of HFD feeding, Ad-Yap/Taz-KO mice showed a strongly reduced expression of the adipocyte-specific lipid droplet protein perilipin-1, accumulation of macrophages, and increased expression of M2 macrophage marker genes including *Arg1*, *Mrc1*, and *IL-10*, whereas expression of M1 marker genes such as *iNOS* was rather decreased (Fig. 3f–h). Furthermore, expression of several adipocyte marker genes, such as adiponectin (*Adipoq*), *Leptin*, *Resistin*, *Glut4*, *Pparγ*, and *Fabp4*, were strongly reduced in the WAT of Ad-Yap/Taz-KO mice (Fig. 3i). Collectively, these data confirm that YAP and TAZ are essential for adipocyte survival during obesity, and that loss of YAP and TAZ leads to adipocyte apoptosis and lipodystrophy.

### YAP and TAZ regulate expression of genes encoding pro- and anti-apoptotic factors in adipocytes

The massive increase in apoptotic adipocyte death in obese mice after loss of YAP/TAZ in adipocytes suggests that YAP/TAZ activation during obesity promotes anti-apoptotic, pro-survival pathways, or inhibits pro-apoptotic pathways. To determine whether YAP/TAZ are involved in regulation of the pro-survival nuclear factor-κB (NF-κB) signaling pathway, we analyzed the phosphorylation of NF-κB in adipose tissue lysates from Ad-Yap/Taz-KO mice fed HFD for 4 weeks but found no obvious difference in phosphorylation of P65 compared to controls (Supplementary Fig. 4a). Deficiency of YAP/TAZ also did not alter tumor necrosis factor-α (TNFα)-induced P65 phosphorylation in primary adipocytes from wild-type and Ad-Yap/Taz-KO mice fed HFD for 4 weeks (Supplementary Fig. 4b). This indicates that YAP/TAZ are not critically involved in NF-κB activation during obesity.

**Fig. 4:**
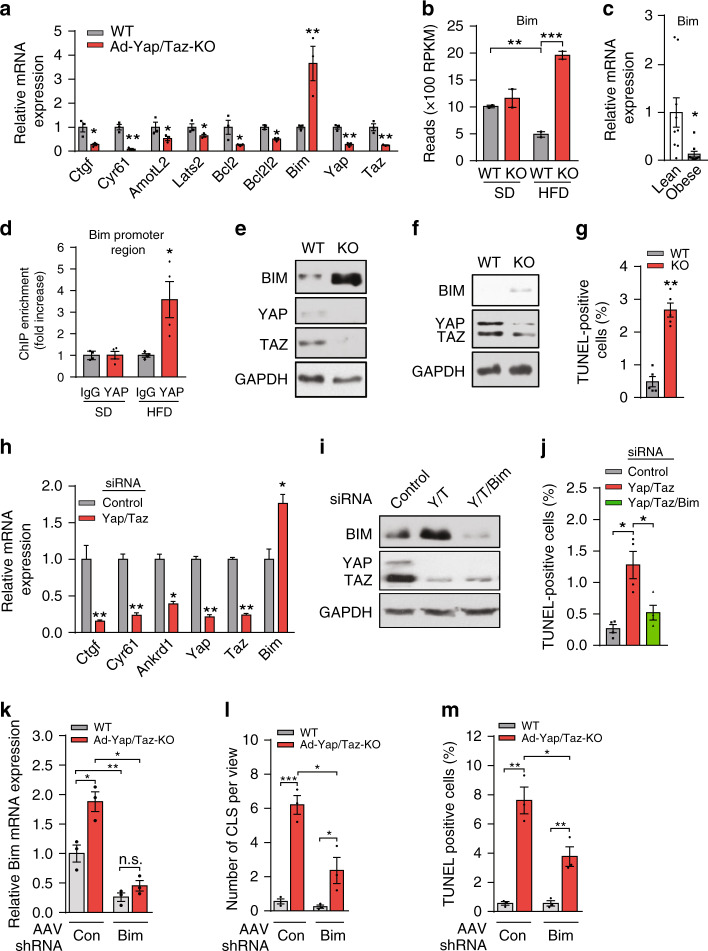
YAP/TAZ promote adipocyte survival by suppression of Bim expression. **a** Quantitative RT-PCR showing mRNA expression of Yap, Taz, and their target genes including pro- and anti-apoptotic factors in white adipocytes isolated from WT and Ad-Yap/Taz-KO mice fed HFD for 4 weeks (*n* = 3 mice per group). **b** RNA-seq data showing expression of Bim (Bcl2l11) in white adipocytes isolated from WT and Ad-Yap/Taz-KO mice fed HFD or standard diet for 4 weeks (*n* = 2 mice per group). **c** Expression of Bim in isolated adipocytes from the vWAT of lean (BMI < 25) and obese humans (BMI > 30) (*n* = 9 in both groups). **d** Chromatin immunoprecipitation (ChIP) PCR of the TEAD-binding site in the Bim promoter region in lysates of vWAT prepared from WT mice fed standard diet (SD) or HFD for 4 weeks using an anti-YAP antibody (#4912, Cell Signaling Technology) (*n* = 4 mice per group). **e**, **f** Western blot analysis showing BIM protein level in white adipocyte isolated from WT and Ad-Yap/Taz-KO mice (KO) fed HFD for 4 weeks (**e**) and in adipocytes differentiated from the SVF of WT and Ad-Yap/Taz-KO mice (KO) (**f**). **g** Apoptotic cell death detected by TUNEL staining of adipocytes differentiated from SVF of WT and Ad-Yap/Taz-KO mice (*n* = 5 mice per group). **h** Quantitative RT-PCR showing mRNA expression of YAP/TAZ target genes in differentiated 3T3-L1 adipocytes treated with control siRNA or siRNA directed against Yap and Taz (*n* = 3). **i**, **j** Differentiated 3T3-L1 adipocytes were treated with control siRNA or siRNA directed against Yap, Taz, and Bim, and western blot analysis was performed to analyze protein levels of BIM, YAP, TAZ, and GAPDH (**i**). In parallel, cells were analyzed by TUNEL staining to determine cell apoptosis (**j**) (*n* = 4). **k** Bim expression in adipocytes isolated from vWAT 4 weeks after injection of adeno-associated virus transducing control shRNA (AAV-Con) or shRNA directed against Bim (AAV-shBim) fed HFD (*n* = 3 mice per group). **l**, **m** Quantification of crown-like structures (CLS) (**l**) and of TUNEL-positive cells (**m**) in sections of vWAT from WT and Ad-Yap/Taz-KO mice 4 weeks after injection of adeno-associated virus transducing control shRNA (AAV-Con) or shRNA directed against Bim (AAV-shBim) and fed HFD (*n* = 3 mice per group, at least 10 sections were analyzed per animal). Data are presented as the mean ± SEM; *p ≤ 0.05 and **p ≤ 0.01 (unpaired Student’s *t*-test in **a**, **c**, **g**, **h** and one-way ANOVA in **b**, **d**, **j**–**m**). Source data are provided as a Source Data file.

To understand the underlying mechanism by which YAP/TAZ modulate adipocyte survival during obesity, we performed expression profiling by RNA sequencing (RNA-seq) and quantitative RT-PCR using total RNA isolated from white adipocytes of Ad-Yap/Taz-KO mice and of wild-type littermates, which had been fed HFD for 4 weeks. As expected, YAP/TAZ target genes, such as *Ctgf*, *Cyr61*, *AmotL2*, and *Lats2*, showed reduced expression in Ad-Yap/Taz-KO adipocytes (Fig. 4a and Supplementary Fig. 4c). In addition, we found that the anti-apoptotic genes *Bcl2* and *Bcl2l2* were downregulated, whereas the pro-apoptotic gene *Bim* was upregulated in the absence of Yap/Taz (Fig. 4a and Supplementary Fig. 4c). Interestingly, in adipocytes from HFD-fed wild-type animals as well as from the vWAT of obese humans, the pro-apoptotic factor BIM (BCL2L11) was downregulated compared to lean controls (Fig. 4b, c), and in chromatin immunoprecipitation (ChIP) assays we found that YAP showed increased binding to a TEAD-binding motif in the promoter region of Bim in adipocytes from HFD-fed mice compared to animals fed an SD (Fig. 4d). The strong upregulation of Bim expression in the absence of YAP/TAZ was confirmed on the protein level in primary adipocytes isolated from HFD-fed Ad-Yap/Taz-KO mice, as well as in adipocytes differentiated from the SVF (stromal vascular fraction) of Ad-Yap/Taz-KO mice in vitro (Fig. 4b, e, f), which also showed increased apoptosis (Fig. 4g). Suppression of Yap and Taz expression in differentiated 3T3-L1 adipocytes also resulted in increased Bim expression and apoptosis (Fig. 4h–j), and small interfering RNA (siRNA)-mediated suppression of Bim expression blocked the increase in apoptosis (Fig. 4i, j). Consistent with this, suppression of Bim expression in epididymal adipocytes of HFD-fed Ad-Yap/Taz-KO mice in vivo by viral transduction of a corresponding short hairpin RNA (shRNA) resulted in a significantly reduced number of crown-like structures and of TUNEL-positive cells (Fig. 4k–m), and was accompanied by a significant decrease in fat loss (Supplementary Fig. 4d). These data indicate that YAP/TAZ suppress expression of the pro-apoptotic gene Bim to promote adipocyte survival.

### TNFα and IL-1β induce YAP/TAZ nuclear translocation and YAP/TAZ transcriptional activity

**Fig. 5:**
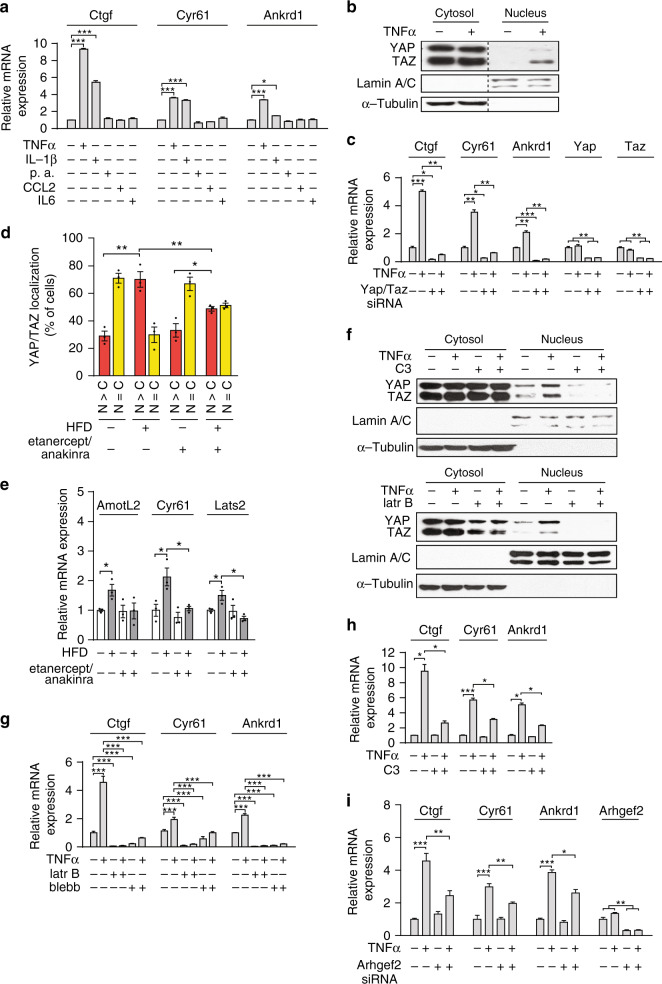
TNFα regulates YAP/TAZ transcriptional activity in adipocytes. **a** Quantitative RT-PCR showing mRNA expression of YAP/TAZ target genes in response to TNFα (1.1 nM), IL-1β (5.9 nM), IL-6 (0.8 nM), CCL2 (1.2 nM), and palmitic acid (p.a., 500 nM) in differentiated 3T3-L1 adipocyte (*n* = 3 per group). **b** Differentiated 3T3-L1 adipocytes were incubated without or with 1.1 nM TNFα for 6 h, then cytosolic and nuclear fractions were analyzed by immunoblotting using antibodies against YAP/TAZ, α-Tubulin (cytoplasmic marker), and Lamin A/C (nuclear marker). The dashed line indicates the margins of a region, which was cut out. **c**, **g**, **h** Differentiated 3T3-L1 adipocytes were treated with control siRNA or siRNA directed against Yap/Taz (**c**) or were pretreated with 2.5 µM latrunculin B (latr B), 20 µM blebbistatin (blebb) for 30 min (**g**), or with 1 µg/ml of C3-exoenzyme (**h**) for 6 h. Thereafter, cells were incubated without or with 1.1 nM TNFα for 8 h and expression of YAP/TAZ target genes was analyzed (*n* = 3). **d** Quantification of YAP/TAZ localization to the nucleus (N) or cytosol (C) in the visceral (vWAT) from standard diet (SD)-fed (*n* = 3) and HFD-fed mice (*n* = 3) treated without or with etanercept and anakinra by intraperitoneal injection. **e** Expression of YAP/TAZ target genes in white adipocyte isolated from the vWAT of mice fed HFD (*n* = 3) or standard diet (SD) and treated without or with etanercept and anakinra (*n* = 3). **f** Differentiated 3T3-L1 adipocytes were preincubated with C3-exoenzyme (C3) or latrunculin B (latr B). Thereafter, cells were incubated without or with 1.1 nM TNFα for 6 h, and cytosolic and nuclear fractions were analyzed by immunoblotting using antibodies against YAP, TAZ, Lamin A/C, and α-Tubulin. **i** Differentiated 3T3-L1 adipocytes were treated with control siRNA or siRNA directed against Arhgef2. Thereafter, cells were incubated in the absence or presence of TNFα (1.1 nM) and expression of YAP/TAZ target genes was determined (*n* = 4). Data are presented as the mean ± SEM; *p ≤ 0.05, **p ≤ 0.01, and ***p ≤ 0.001 (one-way ANOVA). Source data are provided as a Source Data file.

To identify signals in fat tissue that might induce YAP/TAZ activation during obesity, we analyzed whether palmitic acid or several cytokines, including TNFα, interleukin (IL)-1β, CCL2, and IL-6 are able to induce YAP/TAZ target gene expression in 3T3-L1 adipocytes. Quantitative RT-PCR analysis showed that expression of the YAP/TAZ target genes *Ctgf*, *Cyr61*, and *Ankrd1* was induced by TNFα or IL-1β (Fig. 5a). Consistent with this, we detected increased YAP/TAZ nuclear translocation in response to TNFα and IL-1β (Fig. 5b and Supplementary Fig. 5a), and suppression of Yap/Taz expression strongly inhibited TNFα- and IL-1β-induced YAP/TAZ target gene expression (Fig. 5c and Supplementary Fig. 5b). An involvement of TNFα and IL-1β in the activation of YAP and TAZ in mouse adipocytes during obesity could be demonstrated by treating animals with the TNFα inhibitor etanercept and the IL-1β inhibitor anakinra. In HFD-fed mice, etanercept and anakinra significantly reduced HFD-induced nuclear translocation of YAP/TAZ, as well as upregulation of YAP/TAZ target gene expression (Fig. 5d, e). In differentiated adipocytes, TNFα or IL-1β did not change protein or phosphorylation levels of YAP/TAZ (Supplementary Fig. 5c) and knockdown of the Hippo pathway kinase LATS1/2 did not affect induction of YAP/TAZ target gene expression by TNFα or IL-1β (Supplementary Fig. 5d). This indicates that regulation of YAP/TAZ activity by TNFα or IL-1β is independent of the Hippo pathway. Inhibition of RhoA, which was activated by TNFα in 3T3-L1 adipocytes (Supplementary Fig. 5e) by C3-exoenzyme, depolymerization of actin by latrunculin B, and inhibition of myosin II ATPase by blebbistatin attenuated TNFα- and IL-1β-induced nuclear translocation of YAP/TAZ (Fig. 5f and Supplementary Fig. 5f–h), as well as TNFα- and IL-1β-induced YAP/TAZ target gene expression (Fig. 5g, f and Supplementary Fig. 5i, j). To examine which RhoGEF protein is involved in TNFα-induced RhoA activation in adipocytes, we focused on ArhGEF2 (GEF-H1), which has been described to be activated through the TNFα receptor^45^, on ArhGEF11 (PDZRhoGEF), which has been involved in adipocyte metabolism^46^, as well as on the related RhoGEF proteins ArhGEF1 and ArhGEF12 (LARG). Whereas knockdown of ArhGEF1, ArhGEF11, and ArhGEF12 had no effect on TNFα-induced upregulation of YAP/TAZ target genes (Supplementary Fig. 5k), knockdown of ArhGEF2 blocked the TNFα effect (Fig. 5i). This suggests that ArhGEF2- and RhoA-mediated actomyosin contractility is required for TNFα- and IL-1β-induced YAP/TAZ nuclear translocation and subsequent transcriptional activity.

**Fig. 6:**
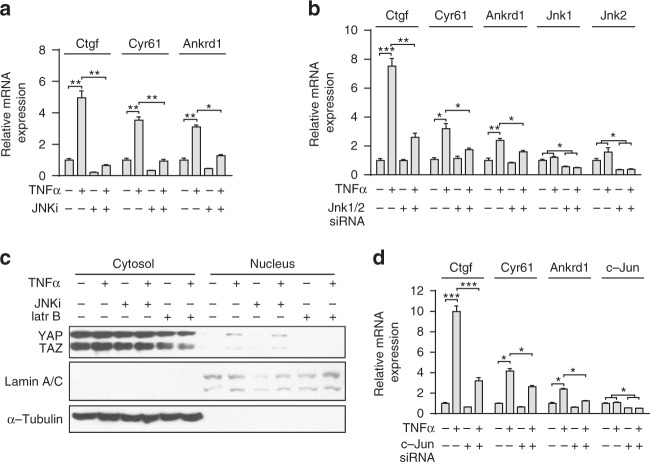
JNK and c-JUN mediate TNFα-induced YAP/TAZ transcriptional activity. **a**–**d** Differentiated 3T3-L1 adipocytes were incubated in the absence or presence of the JNK inhibitor SP600125 (JNKi, 5 µM) (**a**, **c**), latrunculin B) (latr B, 2.5 µM) (**c**), or were treated with control siRNA or siRNA directed against Jnk1 and 2 (**b**) or c-Jun (**d**). Thereafter, cells were incubated in the absence or presence of TNFα (1.1 nM), and expression of YAP/TAZ target genes was determined (*n* = 3) (**a**, **b**, **d**) or cytosolic and nuclear fractions were analyzed by immunoblotting using antibodies against YAP and TAZ, Lamin A/C or α-Tubulin (**c**). Shown are mean values mean ± SEM; *p ≤ 0.05, **p ≤ 0.01, and ***p ≤ 0.001 (one-way ANOVA). Source data are provided as a Source Data file.

To identify additional intracellular signaling pathways activated by TNFα and IL-1β, which are involved in YAP/TAZ activation by both cytokines in adipocytes, we tested the effect of various protein kinase inhibitors on TNFα- and IL-1β-induced YAP/TAZ target gene expression. Whereas inhibition of mitogen-activated protein-kinase kinase, inhibitor of NF-κB-kinase, phosphoinositide-3-kinase, and p38 kinase had no effect (Supplementary Fig. 6a), inhibition of c-Jun N-terminal kinase (JNK) strongly attenuated TNFα- and IL-1β-induced YAP/TAZ target gene expression (Fig. 6a and Supplementary Fig. 6b). A requirement of JNK for TNFα- and IL-1β-induced YAP/TAZ transcriptional activity was validated by knockdown of Jnk, which also blocked TNFα- and IL-1β-induced activation of YAP/TAZ target genes (Fig. 6b and Supplementary Fig. 6c). C3-exoenzyme and latrunculin B did not affect TNFα-induced JNK phosphorylation (Supplementary Fig. 6d), indicating that Rho-mediated signaling is not required for TNFα- and IL-1β-induced JNK activation. Although disruption of the actin cytoskeleton by latrunculin B abolished TNFα- and IL-1β-induced YAP/TAZ nuclear localization, inhibition of JNK activation had no effect (Figs. 5f and 6c, and Supplementary Fig. 5g), indicating that JNK does not regulate YAP/TAZ function on the level of nuclear translocation. As the YAP/TAZ/TEAD complex has been shown to functionally interact with AP-1 that synergistically regulates YAP/TAZ target gene expression^47^, and as JNK phosphorylates and regulates c-Jun, a component of AP-1^48^, we tested whether siRNA-mediated suppression of c-Jun affects TNFα- and IL-1β-induced YAP/TAZ target gene expression. We found that suppression of c-Jun expression attenuated TNFα- and IL-1β-induced YAP/TAZ target gene expression (Fig. 6d and Supplementary Fig. 6e), which indicates that JNK and c-JUN mediate TNFα-induced YAP/TAZ transcriptional activity. Taken together, these data suggest that RhoA-mediated actomyosin contractility is required for TNFα- and IL-1β-induced YAP/TAZ nuclear localization, whereas JNK/c-JUN is essential for YAP/TAZ transcriptional activity.

## Discussion

YAP and TAZ are transcriptional regulators that integrate metabolic pathways, mechanical cues, and various signaling pathways to control different cellular functions including proliferation, differentiation, and cell survival^27–29^. Based on our initial observation that YAP and TAZ, as well as a characteristic YAP/TAZ transcriptional program were activated in mouse and human white adipocytes during the development of obesity, we found that loss of YAP and TAZ expression did not affect adipocyte function in normal healthy mice. However, in animals fed a HFD, loss of YAP and TAZ resulted in lipodystrophy including massive adipocyte death, a compensatory increase in adipogenesis and infiltration by immune cells. This indicates that YAP/TAZ signaling protects mature adipocytes from cell death during obesity.

Given the YAP/TAZ activation in adipocytes during obesity, the question arises how YAP/TAZ are activated during expansion of the adipose tissue. As adipocytes increase in size during obesity, they experience increased mechanical stress due to compressive forces or strain exerted by the extracellular matrix on expanding adipocytes^49,50^. There is also an increase in connective fiber content in obese adipose tissue, which results in an increase in overall rigidity of the adipose tissue and which has been shown to contribute to increased cell death in the obese adipose tissue^51^. In fact, YAP and TAZ activity is controlled by mechanical signals, such as extracellular matrix rigidity, shear stress, and stretching^35,52,53^, and cytoskeletal tension has been linked to YAP/TAZ activity^28,35–37^. A stiff environment favors YAP nuclear localization and activation, whereas attachment to soft substrates increases cytoplasmic retention of YAP^35^. Recent work has shown that integrin adhesion to the extracellular matrix and integrin signaling, as well as RhoA-mediated signaling and actomyosin-driven contractility play important roles in mechanical regulation of YAP/TAZ activity^28,54–58^, which appears to be largely independent of the Hippo pathway^35,59^. In addition, actomyosin-mediated tension has been shown to regulate the thermogenic capacity of brown adipocytes through YAP and TAZ^60^. However, it is not known whether a similar mechanism exists in white adipocytes. Our data show that inhibition of RhoA-mediated signaling, latrunculin B-induced depolymerization of actin, and myosin II ATPase inhibition by blebbistatin reduced cytokine-induced YAP/TAZ activation in differentiated adipocytes, suggesting that increased cytoskeletal tension in obese adipocytes contributes to YAP/TAZ activation during obesity.

The enlargement of the adipose tissue during obesity is accompanied by the development of a chronic low-grade inflammation of the adipose tissue, which includes infiltration of the adipose tissue by macrophages and increased levels of cytokines^13–19^. By testing several mediators that are increased in the obese adipose tissue, we identified TNF-α and IL-1β, which have been shown to be produced by adipocytes and cells of the SVF including infiltrating macrophages in obese mice and humans^61–65^, as potent activators of adipocyte YAP/TAZ. This suggests that inflammatory mediators produced in the obese adipose tissue contribute to adipocyte YAP/TAZ activation during obesity. TNF-α and IL-1β can activate several intracellular signaling pathways including extracellular signal-regulated kinase, IKK/NF-κB-signaling, p38, and JNK^66^. By pharmacological inhibition and siRNA-mediated knockdown, we identified JNK as a critical signaling component in cytokine-induced YAP/TAZ activation in adipocytes. Although inhibition of JNK had no effect on cytokine-induced nuclear translocation of YAP/TAZ, it strongly inhibited cytokine-induced activation of the YAP/TAZ transcriptional program, which we found to also require c-JUN, a component of AP-1^48^. As the YAP/TAZ/TEAD complex has been shown to functionally interact with AP-1 to synergistically regulate target gene expression^47^, we conclude that a similar mechanism is also operating in adipocytes. As a major mechanism mediating cytokine-induced YAP/TAZ nuclear translocation, we identified RhoA-mediated actomyosin contractility, whereas blockade of the Hippo pathway component LATS1/2 had no effect on cytokine-induced YAP/TAZ nuclear translocation. This is consistent with several reports indicating that Rho/actomyosin-mediated nuclear translocation of YAP/TAZ occurs independently of the Hippo pathway^34,35,67^.

Expansion of the adipose tissue during obesity is accompanied by increased mechanical, metabolic, and inflammatory stress, as well as increased adipocyte cell death^68^. As we observed massive apoptotic adipocyte cell death in the absence of YAP/TAZ in mature adipocytes during obesity, activation of YAP/TAZ during obesity may either promote anti-apoptotic, pro-survival pathways or inhibit pro-apoptotic pathways. The inflammatory NF-κB pathway is also important for adipocyte survival during obesity, as loss of signaling components of this pathway results in increased apoptotic death of adipocytes during obesity^24,26^. We could largely exclude that YAP/TAZ have an effect on IKK/NF-κB pro-survival signaling, as loss of YAP/TAZ did not affect TNF-α-induced activation of NF-κB in vitro and as NF-κB activity was not altered in adipocytes from obese Ad-Yap/Taz-KO mice compared to controls.

When analyzing transcriptomic changes in white adipocytes of Ad-Yap/Taz-KO mice fed HFD for 4 weeks compared to wild-type littermates, we observed that the anti-apoptotic genes *Bcl2* and *Bcl2l2* were downregulated, and that the pro-apoptotic gene *Bim* was strongly upregulated in the absence of YAP/TAZ. This is consistent with previous data, which showed that, during embryonic stem cell (ESC) differentiation, YAP attenuates apoptosis primarily through transcriptional upregulation of anti-apoptotic factors, including BCL2, BCL2L1, and MCL-1^69^, as well as with observations, which showed that YAP/TAZ promote cell survival through suppression of the pro-apoptotic protein BIM, whereas YAP/TAZ knockdown increased BIM expression^70^. Similarly, it has been shown that overexpression of YAP or TAZ suppresses BIM expression^71,72^. In differentiated adipocytes in vitro, we could show that increased apoptotic cell death induced by knockdown of YAP/TAZ, which was accompanied by increased BIM expression, could be rescued by knockdown of Bim. Thus, we conclude that increased activation of YAP/TAZ in obese adipocytes suppresses expression of pro-apoptotic BIM and promotes expression of anti-apoptotic factors such as Bcl2 and Bcl2l2, and that these transcriptional regulatory effects contribute to the protective effect of YAP/TAZ against increased adipocyte cell death during obesity. Targeting YAP/TAZ signaling may provide a novel therapeutic strategy to treat obesity by slowing the expansion of the white adipose mass during hypercaloric diet.

## Methods

### Reagents

Tamoxifen (catalog number T5648), collagenase II (catalog number C6885), wortmannin (catalog number W3144), latrunculin B (catalog number 428020), and IL-6 (catalog number I1395) were from Sigma-Aldrich. SC514 was from Santa Cruz Biotechnology (catalog number 354812-17-2). SP600125 (catalog number S5567) and p38 MAPK inhibitor III (catalog number 506148) were obtained from Merck Chemicals GmbH. Exoenzyme C3 transferase was obtained from Cytoskeleton (catalog number CT03-A). Mouse TNFα was purchased from PeproTech (catalog number 315-01 A). IL-1β was from Life Technologies GmbH (catalog number 10139-HNAE). CCL2 (catalog number 479-JE-10) was from Bio-Techne GmbH. The following antibodies were obtained from Cell Signaling Technology: anti-YAP/TAZ (#8418, 1:1000), anti-YAP (#4912, 1:1000), anti-phospho-YAP (Ser127) (#4911, 1:1000), anti-phospho-TAZ (Ser89) (#59971, 1:1000), anti-Lamin A/C (#2032, 1:1000), anti-GAPDH (#2118, 1:3000), anti-phospho-JNK (Thr183/Tyr185) (#9251, 1:1000), anti-IκBα (#4812, 1:1000), anti-P65 (#4764, 1:1000), anti-phospho-P65 (Ser536) (#3033, 1:1000), and anti-BIM (#2933, 1:1000). Anti-TAZ (#HPA007415, 1:1000) and anti-α-Tubulin (#T9026, 1:3000) were from Sigma-Aldrich. Anti-mouse and rabbit horseradish peroxidase (HRP)-conjugated secondary antibodies were from Cell Signaling Technology.

### Animals

Mice carrying floxed alleles of Yap1 (Yap1^tm1a(KOMP)Mbp^) were obtained from the Knockout Mouse Project (KOMP) Repository (KOMP, Davis, California, USA). ESCs carrying a targeted allele of Taz (Wwtr1^tm1a^), which after removal of a cassette flanked by FRT (flippase recognition target) sites allows for Cre-mediated recombination, were purchased from EUCOMM (Helmholtz Zentrum München). ESC clones were injected into C57BL/6 blastocysts and were transferred to pseudopregnant females. Chimeric offspring were bred with C57BL/6 mice to produce heterozygous animals. Germline transmission was confirmed in the F_1_ generation using a PCR genotyping strategy. To delete the selection cassette, mice were crossed to Flp-deleter mice^73^. Adipocyte-specific Yap/Taz-deficient mice (iAd-Yap/Taz-KO) were generated by crossing Yap^flox/flox^;Taz^flox/flox^ mice with Adipoq-CreER^T2^ transgenic mice^41^. For adipocyte pulse-chase experiments, iAd-Yap/Taz-KO and control animals were crossed with the Cre reporter line Gt(ROSA)26Sor^tm4(ACTB-tdTomato,-EGFP)Luo^/J (mT/mG)^43^ obtained from The Jackson Laboratory (JAX stock number 007676). Yap^flox/flox^;Taz^flox/flox^ mice were also mated with Adipoq-Cre mice^44^ to generate Ad-Yap/Taz-KO mice. For induction of Cre-mediated recombination in iAd-Yap/Taz-KO mice, 1 mg of tamoxifen was injected intraperitoneally for 5 consecutive days at the age of 5 weeks. Animals were fed SD (SC, Altromin) or a Western-type HFD containing 30% fat (ssniff) starting from the age of 6 weeks with water ad libitum. Tamoxifen-treated Yap^flox/flox^;Taz^flox/flox^ littermates served as controls. For in vivo experiments, male animals were used at the age of 8–12 weeks if not stated otherwise, whereas adipose tissue from male mice were tested in several in vitro experiments as indicated. Animal care and experimental procedures followed were compliant with ethical regulations and were approved by the local animal welfare authorities and committees (Regierungspräsidia Karlsruhe and Darmstadt).

### Cell culture

Primary white adipocytes and stromal vascular cell fraction from mouse epididymal fat pads and human primary white adipocytes were isolated as described previously^24^. All human samples were collected with the informed consents from the donors following the bioethics and safety regulations. Isolated primary white adipocytes were cultured at 37 °C and 5% CO_2_ in Dulbecco’s modified Eagle’s medium with 10% fetal bovine serum and antibiotic solution. 3T3-L1 preadipocytes (obtained from American Type Culture Collection) were differentiated into mature adipocytes as previously described^74^.

### siRNA-mediated knockdown

A suspension of differentiated 3T3-L1 adipocytes was transfected with siRNA using Lipofectamine RNAiMax transfection reagent (Invitrogen) according to the manufacturer’s instruction and seeded on collagen-coated plates. The targeted sequences of mouse siRNAs directed against RNAs encoding Yap, Taz, Lats1, Lats2, Jnk1, Jnk2, c-Jun, Bim, Rhogef1, Rhogef2, Rhogef11, and Rhogef12 were as follows: Yap, 5′-GCTAGATAAAGAAAGCTTT-3′ and 5′-CCAATAGTTCCGATCCCTT-3′; Taz, 5′-GAATCAGCCTCTGAATCAT-3′ and 5′-GTCTGAAGATCTGATCCCT-3′; Lats1, 5′-GCAACATTCAATTAACCGA-3′; Lats2, 5′-CCATGAAGACTCTCAGGAA-3′; Jnk1, 5′-CAAGAGATTTGTTATCCAA-3′, 5′-GAAGCAAACGTGACAACAA-3′, 5′-GTTCTTATGAAGTGTGTTA-3′, and 5′-TAAATACGCTGGATATAGC-3′; Jnk2, 5′-CCGCAGAGTTCATGAAGAA-3′, 5′-GCGGATCTCTGTGGACGAA-3′, 5′-AAAGAGAGCATGCGATTGA-3′, and 5′-GCATTCAGCTGGTATCATT-3′; c-Jun, 5′-AGCGTTCGTTATCACAATAAA-3′, 5′-GTGCCTACGGCTACAGTAA-3′, and 5′-GTAACCCTAAGATCCTAAA-3′; Bim, 5′-GTGTTTACATGTGGCGTGT-3′ and 5′-GCCAATTTATGGATGATTT-3′; Arhgef1, 5′-TTGGCTGAACTTGATAGCAGA-3′ and 5′-CTCCAGATTGTCGACATCTAA-3′; Arhgef2, 5′-CACCAAGGCCTTAAAGCTCTA-3′ and 5′-ATCAATCTTTATGGACTTCTA-3′; Arhgef11, 5′-CAGAAGTTTATCAGCAGACAA-3′ and 5′-CTGGGAGATTCTACACCTGAA-3′; Arhgef12, 5′-CAGCAGCTTGTTAGCAGAGAA-3′ and 5′-AAGAATTCAAATATTAAATAA-3′.

### Glucose tolerance test

Intraperitoneal glucose tolerance test and insulin tolerance test were performed as described previously^24^. Briefly, after overnight fasting, glucose was administered intraperitoneally at a dose of 1.5 mg glucose per g body weight for HFD-fed mice and 2 mg glucose per g body weight for mice fed SD. Blood glucose levels were measured in blood taken from the tail vein prior to or at the indicated times after glucose administration using a glucometer (Accu-Chek Aviva, Roche). Insulin sensitivity was determined by intraperitoneal injection of 0.75-1 units of insulin per kg body weight and measuring of glucose levels in blood taken from the tail vein before and at the indicated times after insulin injection in mice fasted for 6 h.

### Adipocyte pulse-chase experiments

Adipocyte pulse-chase experiments were performed as described^42^. Mice were treated with 1 mg/d tamoxifen at the age of 5 weeks by intraperitoneal injection for five consecutive days. Mice were then placed on HFD or remained on SD for an 8-week or a 16-week chase period. Inguinal and epididymal WAT were taken from several regions throughout the depot and were analyzed by whole mount confocal microscopy for tdTomato and eGFP expression. Per mouse, at least 300 (sWAT) or 2000 (vWAT) adipocytes were counted from multiple images.

### Administration of adeno-associated virus

Adeno-associated viral (AAV) vectors of serotype 8, carrying a scrambled sequence (AAV-Ctrl) or an shRNA directed against the mouse *Bim* RNA (AAV-Bim-shRNA) were obtained from Vigen Bioscience. For delivery of virus to the epididymal WAT, mice were anesthetized with 2% isoflurane and a laparotomy was performed to expose the epididymal fat pad under a sterile hood. Virus were diluted to 2.5 × 10^10^ viral genomes/ml in sterile phosphate-buffered saline (PBS) and a total amount of 50 μl *Bim* shRNA virus or control virus was injected at five different sites of each fat pad using a 0.3 cm^3^ 31 G insulin syringe.

### Etanercept and anakira treatment in vivo

Mice fed HFD for 4 weeks were injected during the fourth week with 10 mg/kg etanercept and 25 mg/kg anakira intraperitoneally for 7 consecutive days. Afterwards, animals were killed for fat tissue collection and adipocyte isolation, or whole mount tissue staining.

### Histology and immunohistochemistry

Tissues were fixed by paraformaldehyde (4%), dehydrated in an ethanol gradient, and then transferred to xylene solution for embedding in paraffin. Five-micrometer sections were stained with hematoxylin-eosin and images were analyzed with light microscopy. Adipocyte area in sections was determined in more than 100 cells per condition using ImageJ (NIH). Adipocyte number was calculated as described previously^75^. TUNEL assay was performed on paraffin sections (5 µm) of WAT following the instructions of the In Situ Cell Death Detection Kit (Roche Applied Sciences). For immunohistochemical staining, paraffin sections (5 µm) were placed in a microwave oven for heat-induced epitope retrieval. The following antibodies were used: anti-CD68 (Bio-rad, MCA1957, 1:100) and anti-Perilipin-1 (Cell Signaling Technology, 3470, 1:100). CellMask™ Orange plasma membrane stain (Life Technologies, Thermo Fisher Scientific, C10045) was used to visualize the plasma membrane. Whole fat tissue mount for YAP/TAZ localization (using anti-YAP/TAZ antibody #8418, 1:100, Cell Signaling Technology) and adipocyte pulse-chase experiments was performed as described^76^. Immunofluorescence imaging was performed using a Leica TCS SP5 confocal microscope.

### Quantitative RT-PCR and RNA-seq

Total RNA was isolated from adipocytes or fat tissue using the RNeasy lipid tissue Mini Kit (Qiagen, Valencia, CA, USA) and DNA was removed using the QIAGEN RNase*-*Free DNase Set following the manufacturer’s instructions. cDNA was synthesized using ProtoScript® II reverse transcriptase (New England Biolabs). Quantitative RT-PCR was performed using LightCycler 480 Real-Time PCR detection System (Roche Molecular Biochemicals, Mannheim, Germany) according to the manufacturer’s instructions. Relative mRNA expression was normalized to GAPDH. PCR primers are listed under Supplementary Table 1. For RNA-seq, RNA and library preparation integrity were verified with a BioAnalyzer 2100 (Agilent) or LabChip Gx Touch 24 (Perkin Elmer). Twenty nanograms of total RNA was used as starting material for ribosomal depletion with RiboGone-Mammalian (Clontech) followed by library preparation using SMARTer Stranded Total RNA Sample Prep Kit (Clontech). Sequencing was performed on the NextSeq500 instrument (Illumina) using v2 chemistry, resulting in an average of 35 M reads per library with 1 × 75 bp single end setup. The resulting raw reads were assessed for quality, adapter content, and duplication rates with FastQC (Andrews S. 2010, FastQC: a quality control tool for high-throughput sequence data. Available online at: http://www.bioinformatics.babraham.ac.uk/projects/fastqc). Reaper version 15-065 was employed to trim reads after a quality drop below a mean of Q20 in a window of 20 nucleotides^77^. Only reads between 15 and 75 nucleotides were cleared for further analyses. Trimmed and filtered reads were aligned vs. the Ensembl mouse genome version mm10 (GRCm38) using STAR 2.5.2b with the parameter “–outFilterMismatchNoverLmax 0.1” to increase the maximum ratio of mismatches to mapped length to 10%^78^. The number of reads aligning to genes was counted with featureCounts 1.5.1 tool from the Subread package^79^. Only reads mapping at least partially inside exons were admitted and aggregated per gene. Reads overlapping multiple genes or aligning to multiple regions were excluded. Differentially expressed genes were identified using DESeq2 version 1.14.1^80^. Only genes with a minimum fold change of ±1.5 (log2 ± 0.59), a maximum Benjamini–Hochberg corrected *p*-value of 0.05, and a minimum combined mean of 5 reads were deemed to be significantly differentially expressed. The Ensemble annotation was enriched with UniProt data (release 24.03.2017) based on Ensembl gene identifiers^81^.

### Chromatin immunoprecipitation

ChIP analysis was performed as described previously^82^. vWAT from mice fed with SD or HFD for 4 weeks was washed with PBS and crosslinked with 1% formaldehyde for 10 min. Samples were lysated in adipocyte lysis buffer (5 mM PIPES pH 8.0, 85 mM KCl, and 0.5% NP-40) and homogenized using a Retsch MM 300 TissueLyser Mixer Mill. Then, samples were centrifuged (5 min, 700 × *g*, 4 °C), pelleted nuclei were resuspended in SDS lysis buffer (1% SDS, 10 mM EDTA pH 8.1, and 50 mM Tris·HCl pH 8.1) and sheared with a Bandelin Sonopuls hd 2070 (18 pulses, each pulse 10 s at 100% of the sonicator’s amplitude) to generate DNA fragments of 200–1000 bp length. The chromatin was precleared and incubated with YAP antibody (#4912, Cell Signaling Technology) or IgG overnight under rotation. Complexes were pulled down with 20 µl Magnet ChIP A beads (Millipore) and eluted in elution buffer containing 1% SDS and 100 mM NaHCO_3_. Samples were digested with proteinase K, and DNA was extracted using PCR Clean-up kit (Macherey-Nagel). Samples were analyzed by qPCR using primers Bimfw 5′-TGGATAGACAGTTCCTGGCG-3′ and Bimrev 5′-CGGGCACGACAAGCATATAA-3′.

### Western blottings

The nuclear and cytoplasmatic fraction of adipocytes were separated using NE-PER™ Nuclear and Cytoplasmic Extraction Reagents (Thermo Scientific). Mouse fat tissues or adipocytes were collected, snap-frozen in liquid nitrogen and stored at −80 °C. For protein extraction, ~50 mg frozen tissue was homogenized for 2 min at 30 Hz using a Retsch MM 300 TissueLyser Mixer Mill in 200 μl lysis buffer (50 mM Tris pH 7.5, 2 mM EDTA, 2 mM EGTA, 0.5 M mannitol, 1% Triton X-100, as well as phosphatase inhibitors (10 mg/ml leupeptin, pepstatin A, 4-(2-aminoethyl) benzenesulfonyl-fluoride, and aprotinin) and protease inhibitors (PhosSTOP, Roche))^83^. Extracts were spun down, and fat layer and cell debris were removed. Proteins were separated by SDS-polyacrylamide gel electrophoresis and analyzed by immunoblotting using the indicated antibodies. For detection, Pierce^TM^ ECL Western Blotting Substrate (Thermo Scientific) or Immobilon Western Chemiluminescent HRP Substrate (Merck) were used.

### Study approval

The work on human adipocyte samples was approved by the ethical committee of Xi’an Jiaotong University (XJTU2018-249 and XJTU2019-12) and conforms to the guidelines of the 2000 Helsinki declaration. Written informed consent was obtained from all subjects before their participation. All procedures of animal care and use in this study were approved by the local animal welfare authorities and committees (Regierungspräsidia Karlsruhe and Darmstadt).

### Statistics

Trial experiments or experiments done previously were used to determine sample size with adequate statistical power. Samples were excluded in cases where RNA/cDNA quality or tissue quality after processing was poor (below commonly accepted standards). Animals were excluded from experiments if they showed any signs of sickness. The investigator was blinded to the group allocation and during the experiment. Data represent biological replicates. In all studies, comparison of mean values was conducted with unpaired, two-tailed Student’s *t*-test or one-way or two-way analysis of variance with Bonferroni’s post hoc test. In all analyses, statistical significance was determined at the 5% level (*P* < 0.05). Depicted are mean values ± SEM as indicated in the figure legends. Statistical analysis was performed with Prism5 or Prism6 (GraphPad), or Excel (Microscoft) software.

### Reporting summary

Further information on research design is available in the Nature Research Reporting Summary linked to this article.

## Supplementary information

Supplementary Information

Reporting Summary

## Data Availability

The RNA-seq data are available in GEO repository under the accession GSE138911. Source data are provided with this paper.

## Source data

Source Data
