# Supplementary material for: YAP and TAZ protect against white adipocyte cell death during obesity
Source: Nat Commun. 2020 Oct 28;11:5455. doi: 10.1038/s41467-020-19229-3 (PMC7595161; doi:10.1038/s41467-020-19229-3)

Supplementary information

YAP and TAZ protect against white adipocyte cell death during obesity

(Wang et al.)

Supplementary Figures and Legends

Suppl. Fig. 1

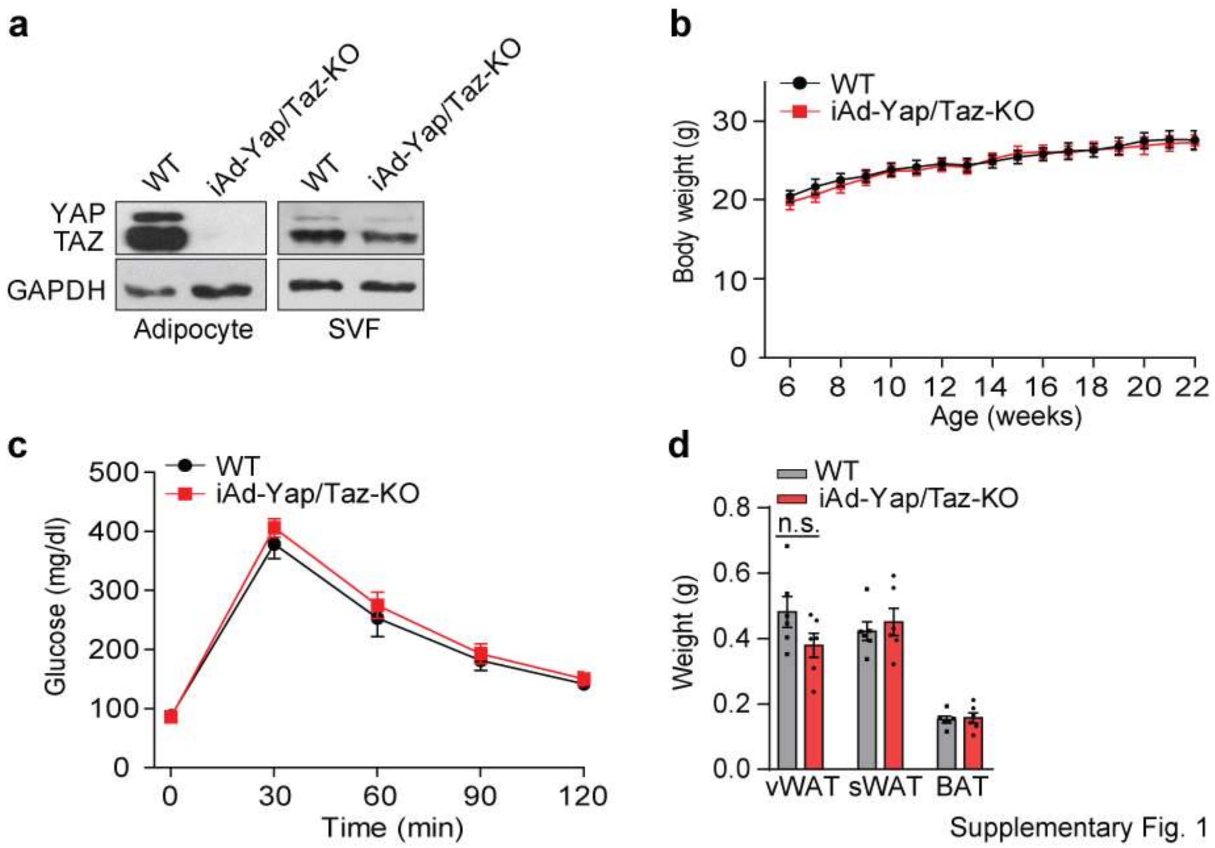

Supplementary Fig. 1. Validation and analysis of iAd-Yap/Taz-KO fed standard diet. (**a**) Western blot showing protein level of YAP and TAZ in isolated adipocytes and the stromal vascular fraction (SVF) of vWAT from wild-type and iAd-Yap/Taz-KO mice 1 week after tamoxifen induction. (**b-d**) Body weight development (b), glucose tolerance (c) and weight of vWAT, sWAT and BAT (brown adipose tissue) (d) in wild-type (n=6) and iAd-Yap/Taz-KO mice (n=6) fed a standard diet. Shown are mean values ± s.e.m.; n.s., not significant. (Two-way ANOVA in **b** and **c** and unpaired Student's *t*-test in **d**).

Suppl. Fig. 2

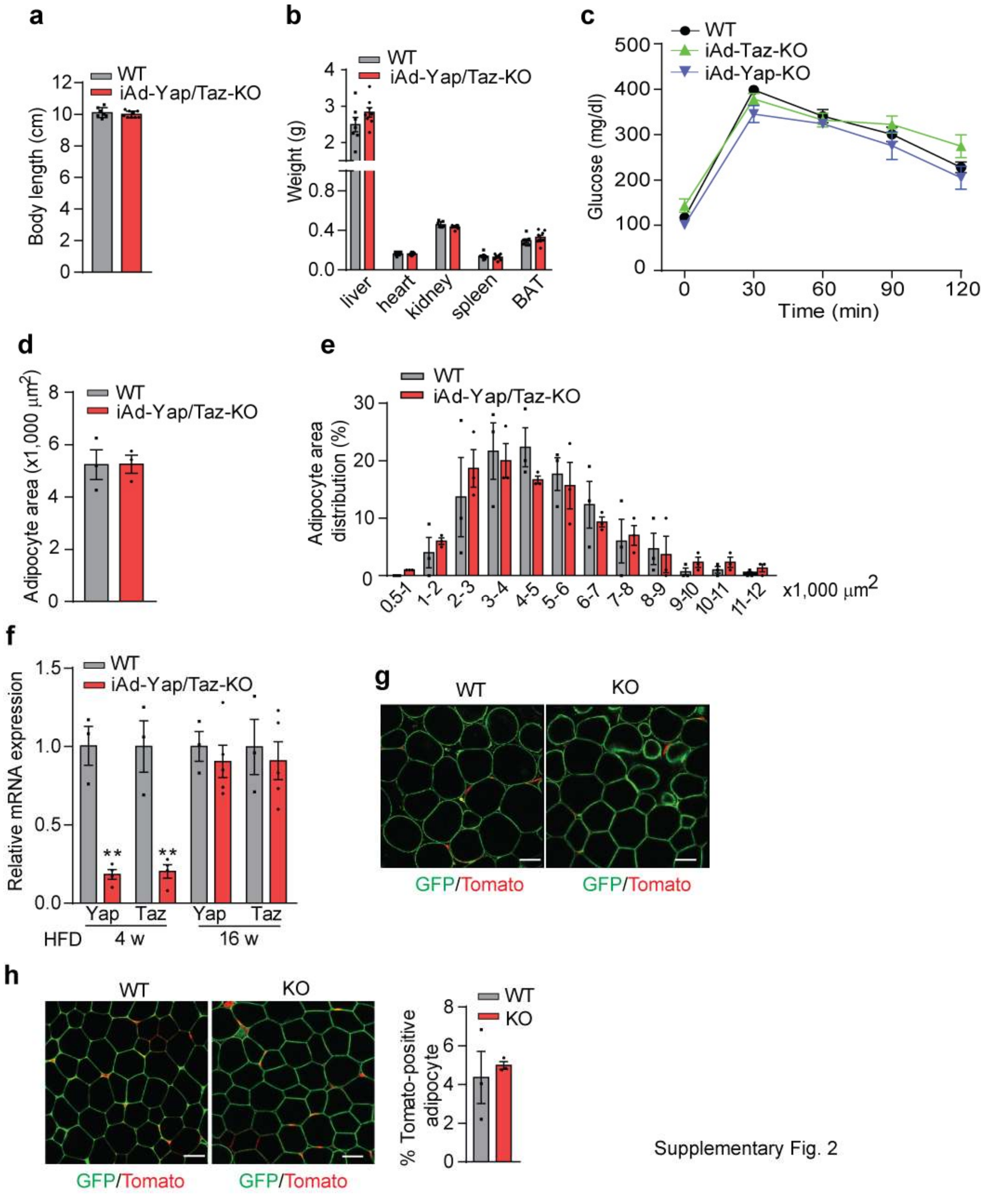

Supplementary Fig. 2. Phenotypical analysis of iAd-Yap/Taz-KO mice fed HFD. (**a**) Body length of wild-type (n=7) and iAd-Yap/Taz-KO mice (n=9) fed HFD for 16 weeks. (**b**) Weight of liver, heart, kidney, spleen and interscapular brown adipose tissue (BAT) from wild-type (n=7) and iAd-Yap/Taz-KO (n=8) mice fed HFD for 16

weeks. (**c**) Intraperitoneal glucose tolerance in wild-type (n=6), iAd-Yap-KO (n=6) and, iAd-Taz-KO mice (n=6). (**d**,**e**) Average visceral adipocyte area (d) and adipocyte area distribution in sections of the vWAT (e) of wild-type and iAd-Yap/Taz-KO mice fed HFD for 16 weeks (n=3 mice per group in d and e; at least 10 sections were analyzed per animal). (**f**) Quantitative RT-PCR showing mRNA expression of Yap and Taz in isolated epididymal adipocyte from wild-type (n=3) and iAd-Yap/Taz-KO (n=4-5) mice fed HFD for 4 and 16 weeks. (**g**,**h**) Representative images of adipocyte tracing in vWAT of Adipoq-CreER^{T2};mT/mG;Yap$^{flox/flox}$;Taz$^{flox/flox}$ mice (KO) and Adipoq-CreER^{T2};mT/mG mice (WT) after tamoxifen treatment and 1 week (g) and 8 weeks (h) of standard diet. The bar diagram in (h) shows the quantification of Tomato positive adipocyte in vWAT of mice fed a standard diet for 8 weeks (n=3 mice per group). Scale bars: 50 µm. Data are presented as the mean ± s.e.m.; **, $p \leq 0.01$ (ANOVA or unpaired Student's *t*-test).

Suppl. Fig. 3

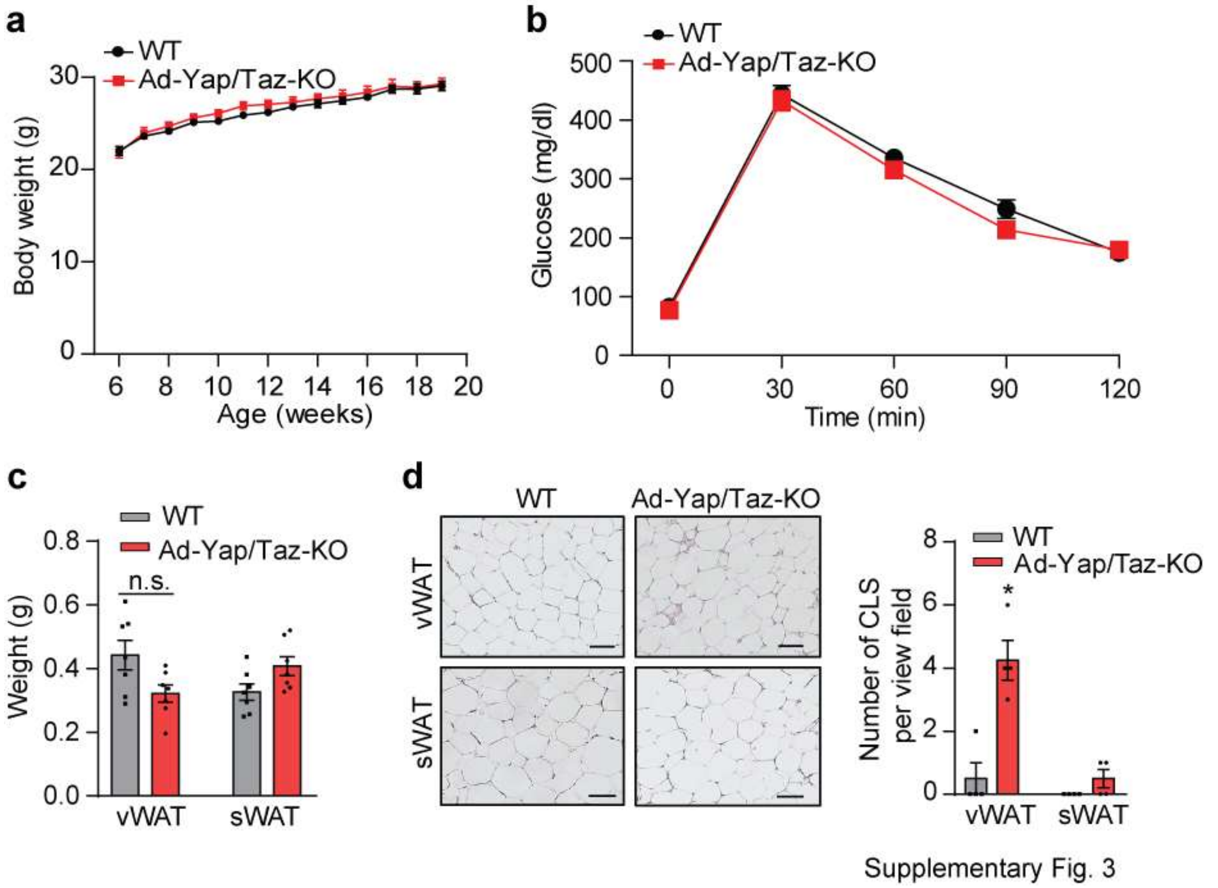

Supplementary Fig. 3. Analysis of Ad-Yap/Taz-KO mice fed standard diet. (**a-c**) Body weight development (a), intraperitoneal glucose tolerance (b) and weight of vWAT and sWAT (c) at an age of 14 weeks of wild-type (WT) (n=6-7) and Ad-Yap/Taz-KO mice (n=5-7). (**d**) H&E-stained epididymal vWAT and sWAT sections from WT and Ad-Yap/Taz-KO mice fed standard diet for 14 weeks. Scale bar: 50 µm. The bar diagram shows the number of crown-like structures (CLS) in vWAT and sWAT from WT (n=3) and Ad-Yap/Taz-KO (n=3) mice. Data are presented as the mean ± s.e.m.; *, $p \leq 0.05$ and n.s., not significant (Two-way ANOVA in **a** and **b** or unpaired Student's *t*-test in **c** and **d**).

Suppl. Fig. 4

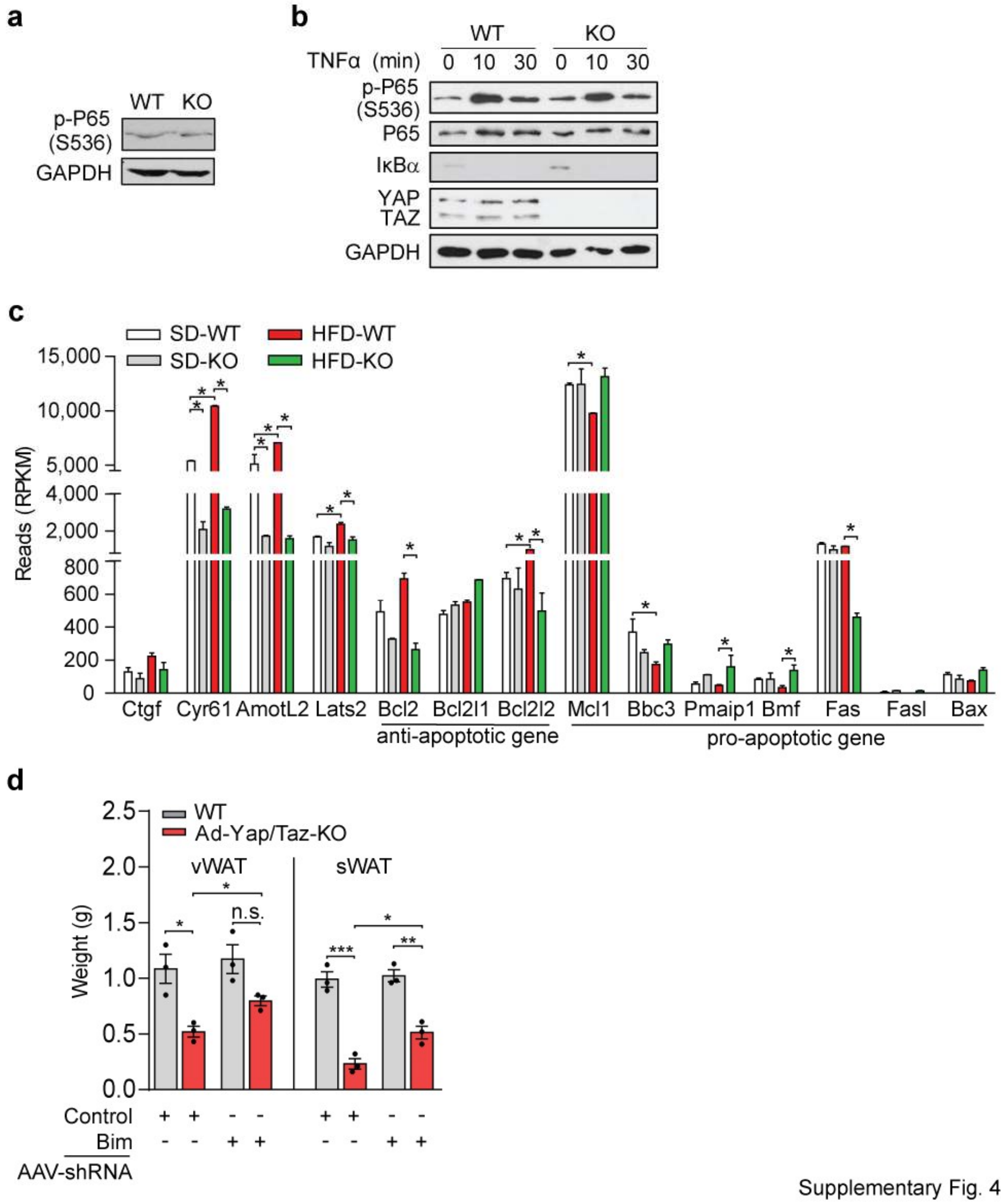

Supplementary Fig. 4. NF-κB activity and gene expression in Ad-Yap/Taz-KO mice. (a) Western blot analysis of P65 phosphorylation levels in vWAT lysates from wild-type (WT) and Ad-Yap/Taz-KO mice (KO) fed HFD for 4 weeks. (b) Western blot analysis of p65 phosphorylation in response to TNFα in adipocytes isolated from vWAT of wild-type (WT) and Ad-Yap/Taz-KO mice fed HFD for 4 weeks. (c) *RNA-seq data showing the* expresion of YAP/TAZ target genes and some pro- and anti-apoptotic genes in adipocytes isolated from vWAT of wild-type (WT, n=2) and Ad-Yap/Taz-KO mice (KO,

n=2) fed standard diet (SD) or HFD for 4 weeks. RPKM: reads per kilobase per million mapped reads. (**d**) Weight of vWAT and sWAT from WT and Ad-Yap/Taz-KO mice 4 weeks after injection of adeno-associated virus transducing control shRNA (AAV-Con) or shRNA directed against Bim (AAV-shBim) and fed HFD (n=3 mice per group). Data are presented as the mean ± s.e.m.; *, $p \leq 0.05$, **, $p \leq 0.01$ and n.s., not significant (unpaired Student's *t*-test in **c** or one-way ANOVA in **d**).

Suppl. Fig. 5

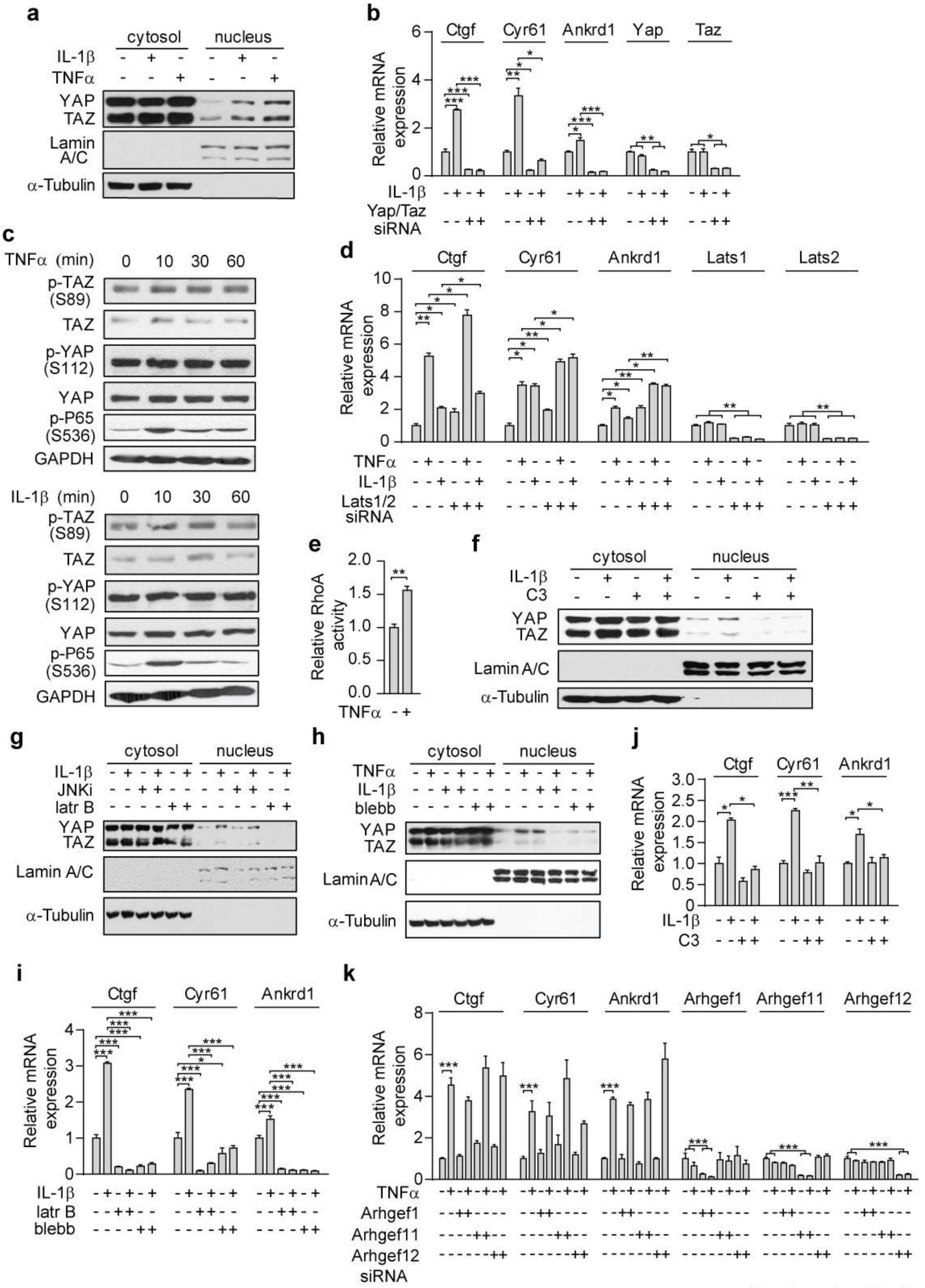

a
cytosol
nucleus
IL-1β
TNFα
YAP
TAZ
Lamin A/C
α-Tubulin
b
Ctgf
Cyr61
Ankrd1
Yap
Taz
Relative mRNA expression
IL-1β
Yap/Taz siRNA
c
TNFα (min)
0
10
30
60
p-TAZ (S89)
TAZ
p-YAP (S112)
YAP
p-P65 (S536)
GAPDH
IL-1β (min)
d
Ctgf
Cyr61
Ankrd1
Lats1
Lats2
Relative mRNA expression
TNFα
IL-1β
Lats1/2 siRNA
e
Relative RhoA activity
TNFα
f
cytosol
nucleus
IL-1β
C3
YAP
TAZ
Lamin A/C
α-Tubulin
g
cytosol
nucleus
IL-1β
JNKi
latr B
YAP
TAZ
Lamin A/C
α-Tubulin
h
cytosol
nucleus
TNFα
IL-1β
blebb
YAP
TAZ
Lamin A/C
α-Tubulin
j
Ctgf
Cyr61
Ankrd1
Relative mRNA expression
IL-1β
C3
i
Ctgf
Cyr61
Ankrd1
Relative mRNA expression
IL-1β
latr B
blebb
k
Ctgf
Cyr61
Ankrd1
Arhgef1
Arhgef11
Arhgef12
Relative mRNA expression
TNFα
Arhgef1
Arhgef11
Arhgef12
siRNA

Supplementary Fig. 5. Effect of IL-1β on YAP/TAZ activity. (**a**) Western blots of cytosolic and nuclear fractions of differentiated 3T3-L1 adipocytes activated by TNFα (1.1 nM) and IL-1β (5.9 nM). Membranes were probed with antibodies against YAP/TAZ, α-Tubulin and Lamin A/C. (**b**,**d**,**i**,**j**,**k**) Differentiated 3T3-L1 adipocytes were treated with control siRNA or siRNA directed against Yap and Taz (b) (n=3) or Lats1 and 2 (d) or the by actin-disrupting agent latrunculin B (latr B) or the myosin II inhibitor blebbistatin (blebb) (n=3) (i) or the Rho GTPase inhibitor C3-exoenzyme (n=3) (j) or control siRNA or siRNA directed against Arhgef1, Arhgef11, Arhgef12 (k). Thereafter, cells were incubated in the absence or presence of 5.9 nM IL-1β (b,d,i,j) or 1.1 nM TNFα (d, k) for 8 hours, and expression of YAP/TAZ target genes was determined. (**c**) Western blot analysis of YAP and TAZ and their phosphorylation level in response to 5.9 nM IL-1β or 1.1 nM TNFα in differentiated 3T3-L1 adipocyte. (**e**) Effect of TNFα (1.1 nM) on RhoA activity in differentiated 3T3-L1 adipocyte (n=3). (**f-h**) Differentiated 3T3-L1 adipocytes were preincubated with C3-exoenzyme (C3, 1 µg/ml) (f), the JNK-inhibitor SP600125 (JNKi, 5 µM) (g), latrunculin B (latr B, 2.5 µM) (h) or blebbistatin (blebb, 20 µM) (h). Thereafter, cells were incubated in the absence or presence of 5.9 nM IL-1β (f-h) or 1.1 nM TNFα (h), and cytosolic and nuclear fractions were analyzed by immunoblotting using antibodies against YAP/TAZ, α-Tubulin and Lamin A/C. Shown are mean values ± s.e.m.; *, $p \leq 0.05$; **, $p \leq 0.01$ (ANOVA).

Suppl. Fig. 6

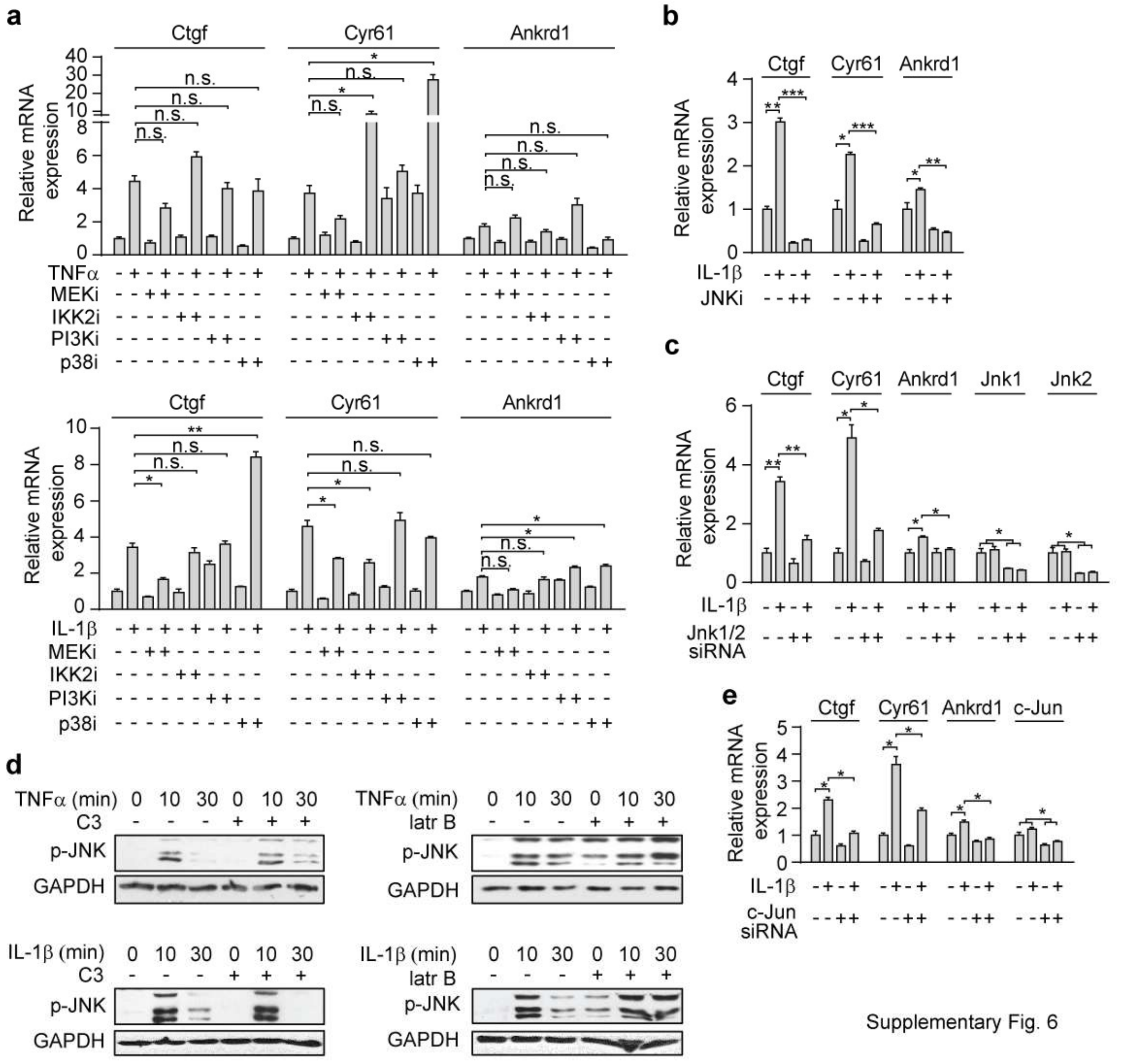

Supplementary Fig. 6. Role of JNK and c-Jun in IL-1β-induced activation of YAP/TAZ transcriptional activity in adipocytes. (**a-c**,**e**) Differentiated 3T3-L1 adipocytes were pre-incubated with the MEK-inhibitor PD98059 (10 µM), IKK2 inhibitor SC-514 (10 µM), PI3K-inhibitor wortmannin (1 µM), p38-inhibitor SB253580 (10 µM) (a) or the JNK-inhibitor SP600125 (5 µM) (b) or were incubated with control siRNA or siRNA directed against Jnk1 and 2 (c) or c-Jun (e). Thereafter, cells were incubated in the absence or presence of 1.1 nM TNFα (a) or IL-1β (b,c,e), and expression of YAP/TAZ target genes was determined (n=3). (**d**) Differentiated 3T3-L1 adipocytes

were pretreated with C3-exoenzyme (C3) or latrunculin B (latr B) and were then incubated with TNFα (1.1 nM) or IL-1β (5.9 nM) for the indicated time periods. Thereafter, whole cell lysates were analyzed by immunoblotting using antibodies against phosphorylated JNK (p-JNK (Thr183/Tyr185)) and GAPDH. Data are presented as mean values ± s.e.m.; *, $p \leq 0.05$; **, $p \leq 0.01$; ***, $p \leq 0.001$ (ANOVA).

Supplementary Table 1 Sequence of qPCR primers (Sigma-Aldrich) used in the study

Gene Symbol	Spieces	forward	reverse
Yap	human	GACATCTTCTGGTCAGAGATACTTCTT	GGGGCTGTGACGTTCATC
Taz	human	CAGCAATGTGGATGAGATGG	TGGGGATTGATGTTCATGG
Cyr61	human	CCAGTGTACAGCAGCCTGAA	GGCCGGTATTTCTTCACACTC
Ctgf	human	ACATTAGTACACAGCACCAGAATGT	GCTATCTGATGATACTAACCTTTCTGC
Lats2	human	AACTGGTGAACGCAGGATG	CCCATCTTGCTGATGTACTCC
AmotL2	human	AGGCTGCAGAGAGACAATGAG	CTCAGAGAGCCGCTGGATT
Bim	human	CAGACAGCAGGTCTCAGGAAG	AAAAATACCCATAAGCGGATCA
Ctgf	mouse	TGACCTGGAGGAAAACATTAAGA	AGCCCTGTATGTCTTCACACTG
Ankrd1	mouse	GCTGGAGCCCAGATTGAA	CTCCACGACATGCCCAGT
Cyr61	mouse	CCCTTCTCCACTTGACCAGA	CACTTGGGTGCCTCCAGA
Yap	mouse	CCTTTGAGATCCCTGATGATG	GCCATGTTGTTGTCTGATCG
TAZ	mouse	GCCACTGGCCAGAGATACTT	GACGGGTGGAGGTTCACAT
CD68	mouse	GACACTTCGGGCCATGTT	GAGGAGGACCAGGCCAAT
F4/80	mouse	GGAGGACTTCTCCAAGCCTATT	AGGCCTCTCAGACTTCTGCTT

iNOS	mouse	TGGCCACCAAGCTGAACT	TTCATGATAACGTTTCTGGCTCT
Il1b	mouse	AGTTGACGGACCCCAAAAG	TTTGAAGCTGGATGCTCTCAT
IL10	mouse	CAGAGCCACATGCTCCTAGA	TGTCCAGCTGGTCCTTTGTT
Cd11c	mouse	GAGCCAGAACTTCCCAACTG	TCAGGAACACGATGTCTTGG
Mcp1	mouse	CATCCACGTGTTGGCTCA	GATCATCTTGCTGGTGAATGAGT
Tnfα	mouse	TCTTCTCATTCCTGCTTGTGG	GGTCTGGGCCATAGAACTGA
Arg1	mouse	GAATCTGCATGGGCAACC	GAATCCTGGTACATCTGGGAAC
Mrc1	mouse	CCACAGCATTGAGGAGTTTG	ACAGCTCATCATTTGGCTCA
Mrc2	mouse	CCCCAAACTCCGACACTG	GGGCCTGGATCCAACTCT
CD163	mouse	GGATGTCGGTGTGATTTGCT	CATCTGGACACTCCATCCACT
IL1α	mouse	TTGGTTAAATGACCTGCAACA	GAGCGCTCACGAACAGTTG
Cd11b	mouse	CAATAGCCAGCCTCAGTGC	GAGCCCAGGGGAGAAGTG
Ifng	mouse	GGAGGAACTGGCAAAAGGAT	TTCAAGACTTCAAAGAGTCTGAGG
Irs1	mouse	CTATGCCAGCATCAGCTTCC	TTGCTGAGGTCATTTAGGTCTTC
Irs2	mouse	TCCAGGCACTGGAGCTTT	GGCTGGTAGCGCTTCACT
Insr	mouse	TCTTTCTTCAGGAAGCTACATCTG	TGTCCAAGGCATAAAAAGAATAGTT

Glut1	mouse	GGATCCCAGCAGCAAGAAG	CCAGTGTTATAGCCGAACTGC
Fabp4	mouse	GGATGGAAAGTCGACCACAA	TGGAAGTCACGCCTTTCATA
Glu4	mouse	GACGGACACTCCATCTGTTG	GCCACGATGGAGACATAGC
CD36	mouse	TTGAAAAGTCTCGGACATTGAG	TCAGATCCGAACACAGCGTA
Adipoq	mouse	GGAGAGAAAGGAGATGCAGGT	CTTTCCTGCCAGGGGTTC
Restin	mouse	AACAAGACTTCAACTCCCTGTTTC	AGACTGCTGTGCCTTCTGG
Leptin	mouse	CAGGATCAATGACATTTCACACA	GCTGGTGAGGACCTGTTGAT
Bim	mouse	TTCCACTTGGATTCACACCA	CTTGGCCATTTGGTCTTTTT
Lats1	mouse	TCCACAGATGTTTCAGGATTTG	GAAGAGCTTGAATAACCATGTCC
Lats2	mouse	GAGGTGCTTCTCCGCAAA	AGCATCTCAAAGAGAATCACACC
Jnk1	mouse	TCTCCAGCACCCATACATCA	TGCTCCCTCTCATCTAACTGC
Jnk2	mouse	GGTATGACCCCGCTGAAG	GCATGCTCTCTTTCTTCCAACT
c-Jun	mouse	TTTCTCACCAACTGCTTGGA	CCAAATGCTCCCCAAAATAC
Arhgef1	mouse	CCTGGAACTGGAACCAGAAG	GACTGAGCAGGGTGTCTGG
Arhgef2	mouse	CCAACAGTTCATCCGGAAA	TGAGCACAGGGTATTTGGTG
Arhgef11	mouse	CAGCCAGAGAACATGTGAAGG	TTCTGTCCCTGAGTCCAAGC

Arhgef12	mouse	CCGGTCTTCGTACAATCTGTC	TCACCTGTTTGTACTCCAGCA

Supplementary materials-Uncropped WB photos
Wang et al

Fig. 4e

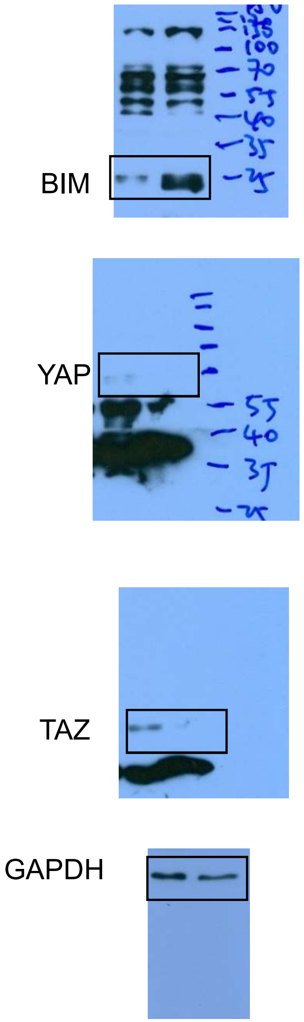

Fig. 4f

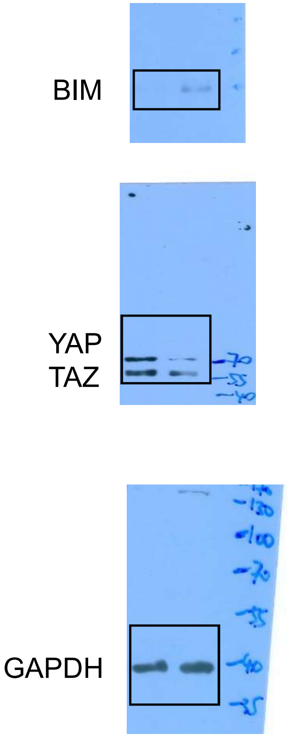

Fig. 4i

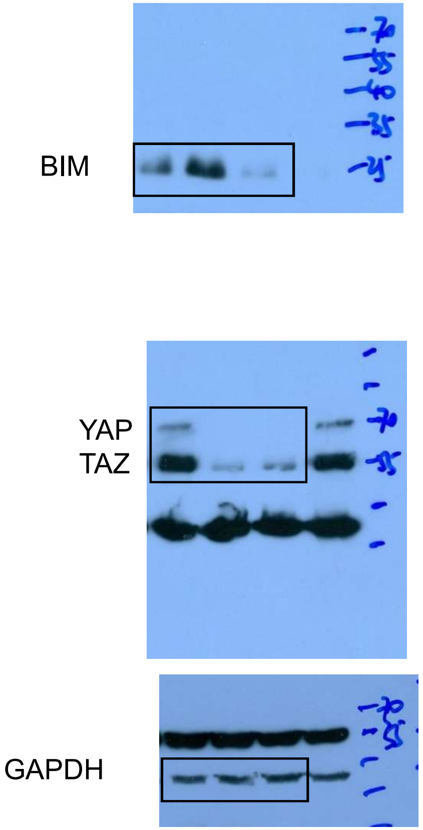

Fig. 5B

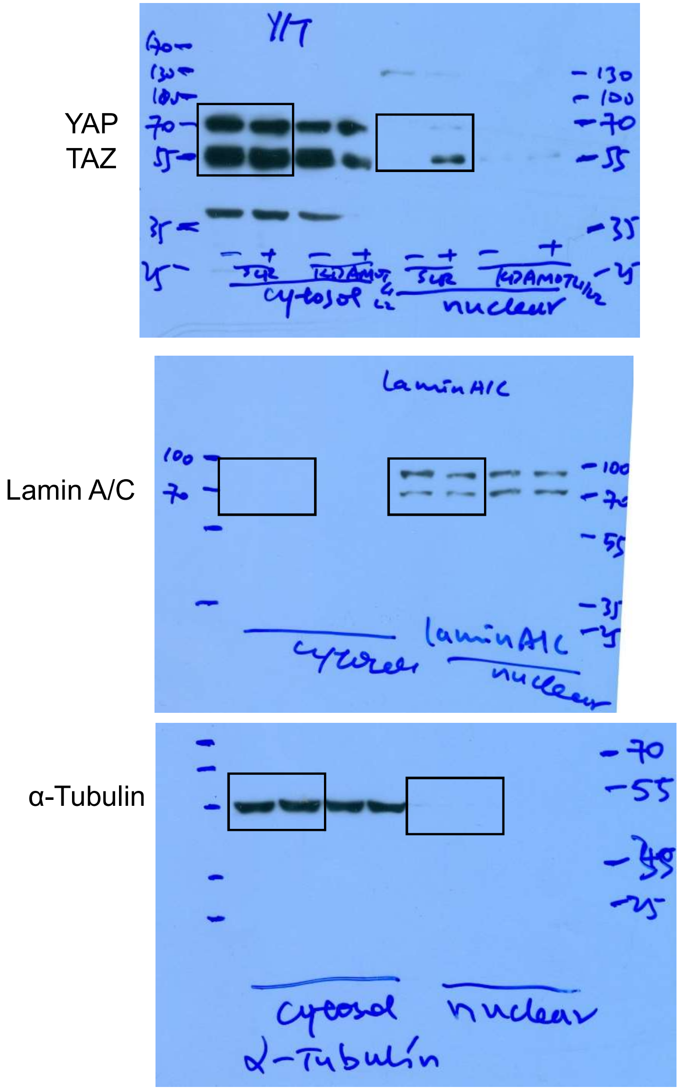

Fig. 5f

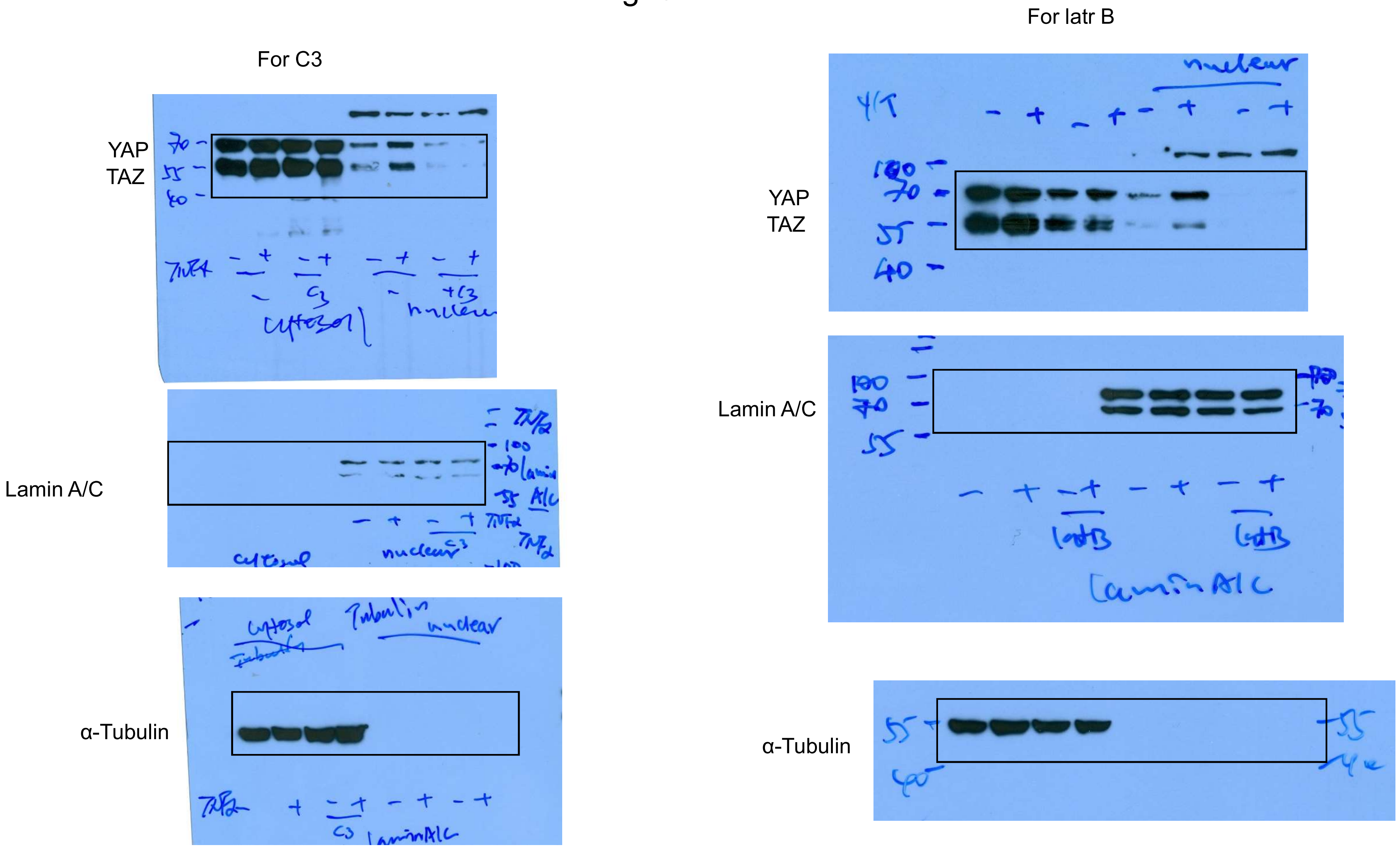
For C3
YAP
TAZ
Lamin A/C
α-Tubulin
For latr B
YAP
TAZ
Lamin A/C
α-Tubulin

Fig. 6c

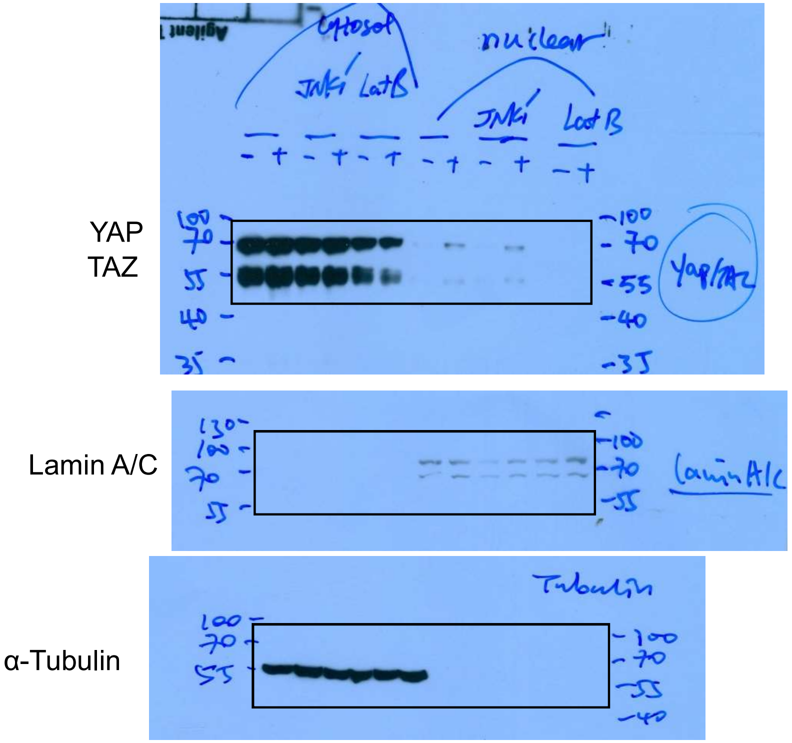

Supplementary Fig. 1a

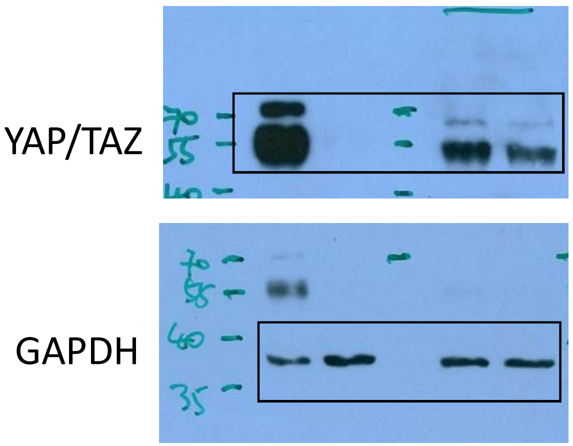

Supplementary Fig. 4a

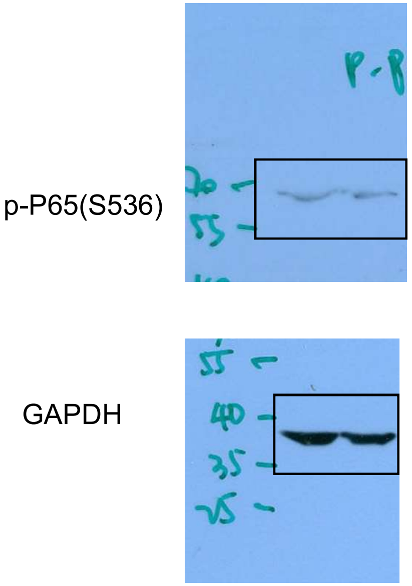

Supplementary Fig. 4b

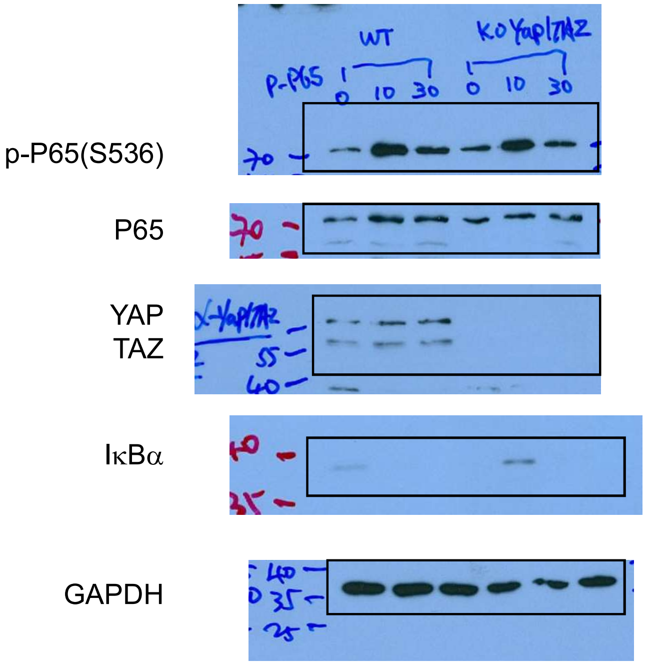

Supplementary Fig. 5a

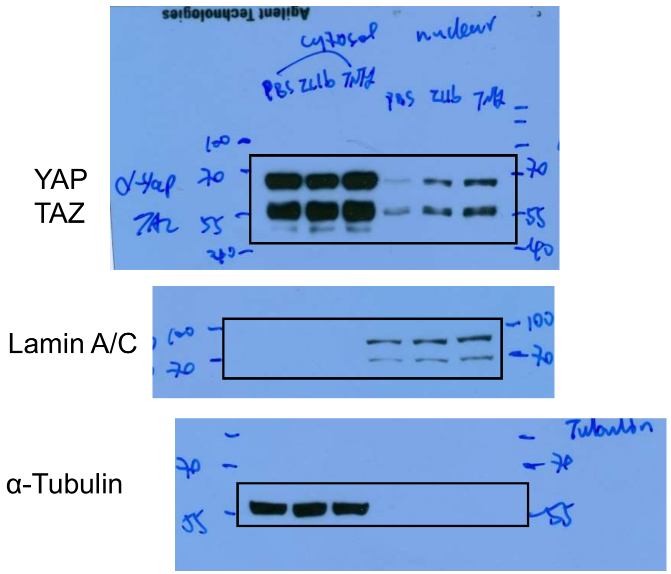

Supplementary Fig. 5c

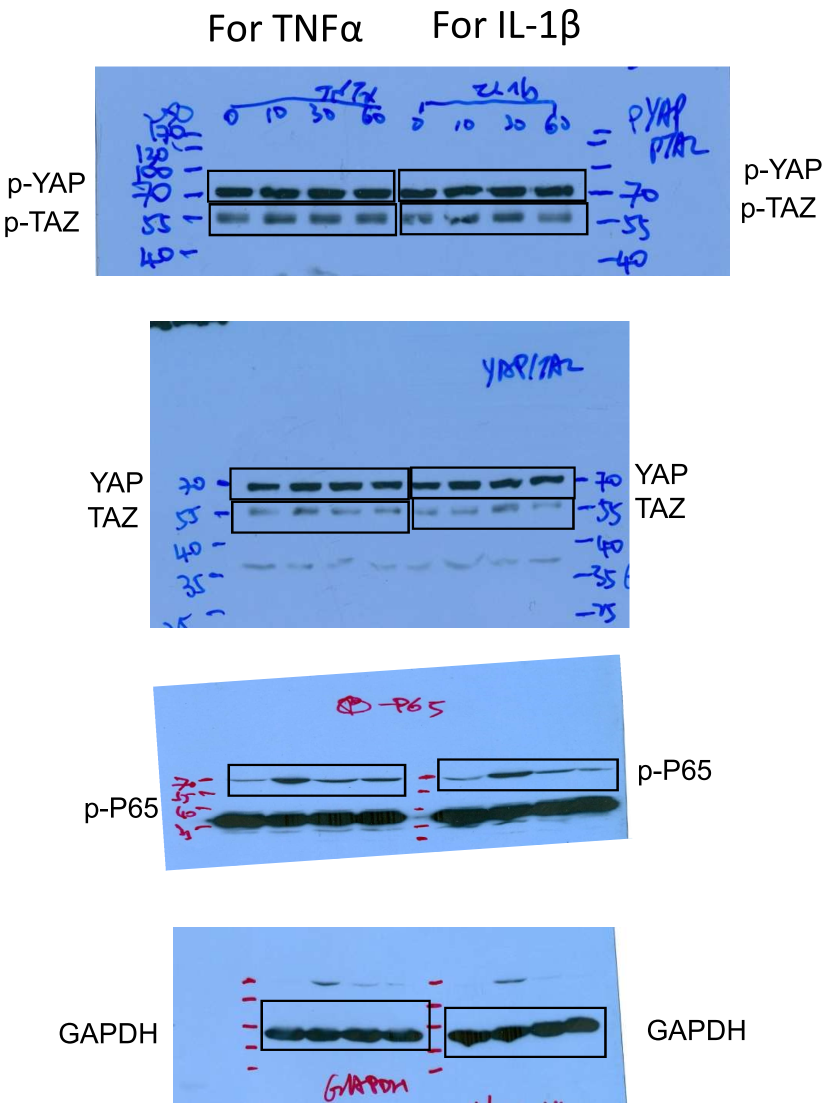

Supplementary Fig. 5f

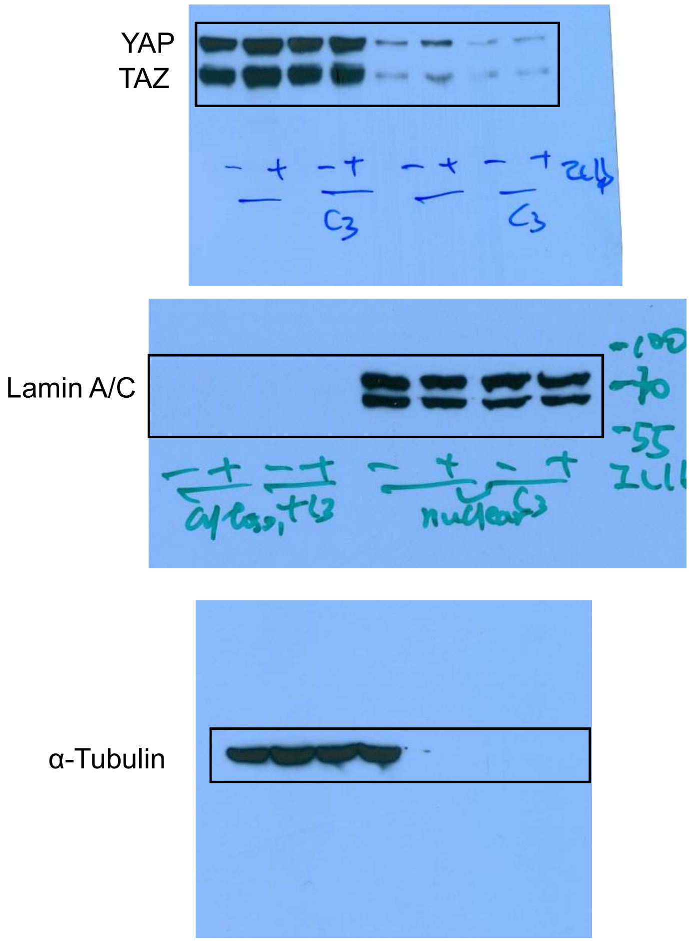

Supplementary Fig. 5g

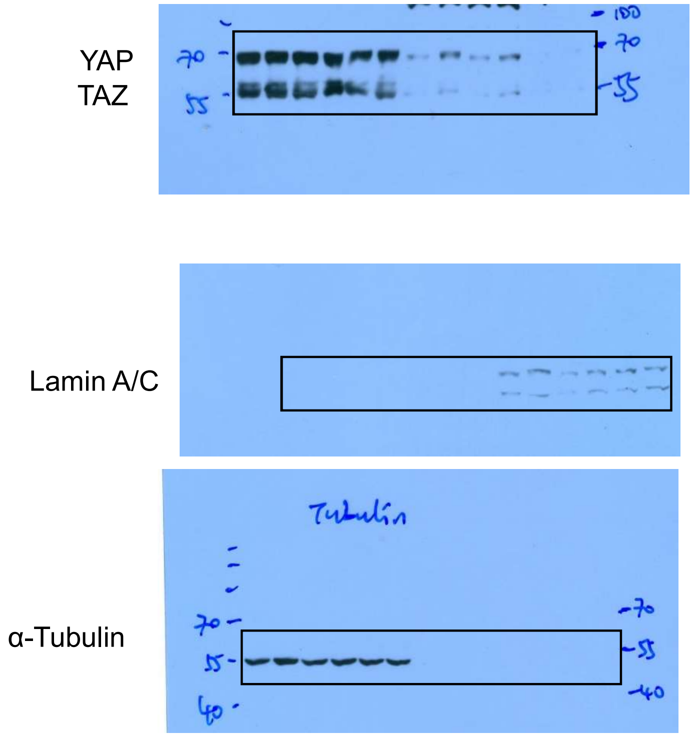

Supplementary Fig. 5h

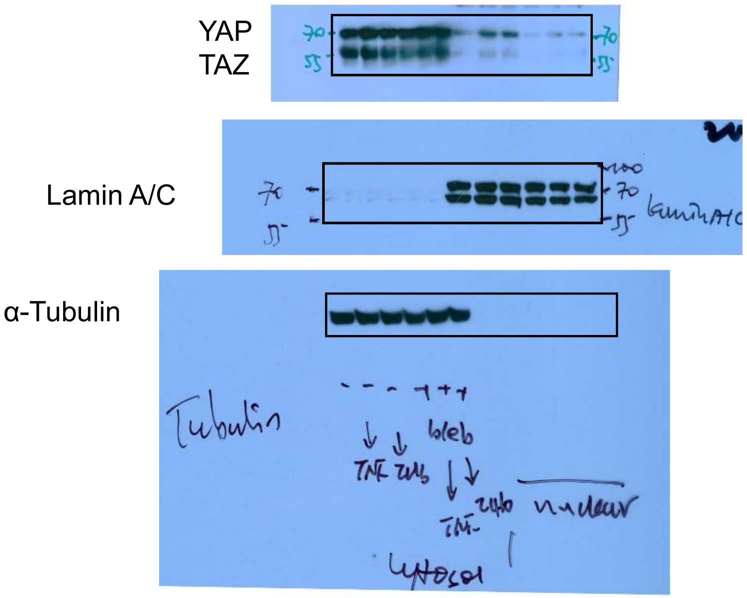

Supplementary Fig. 6d

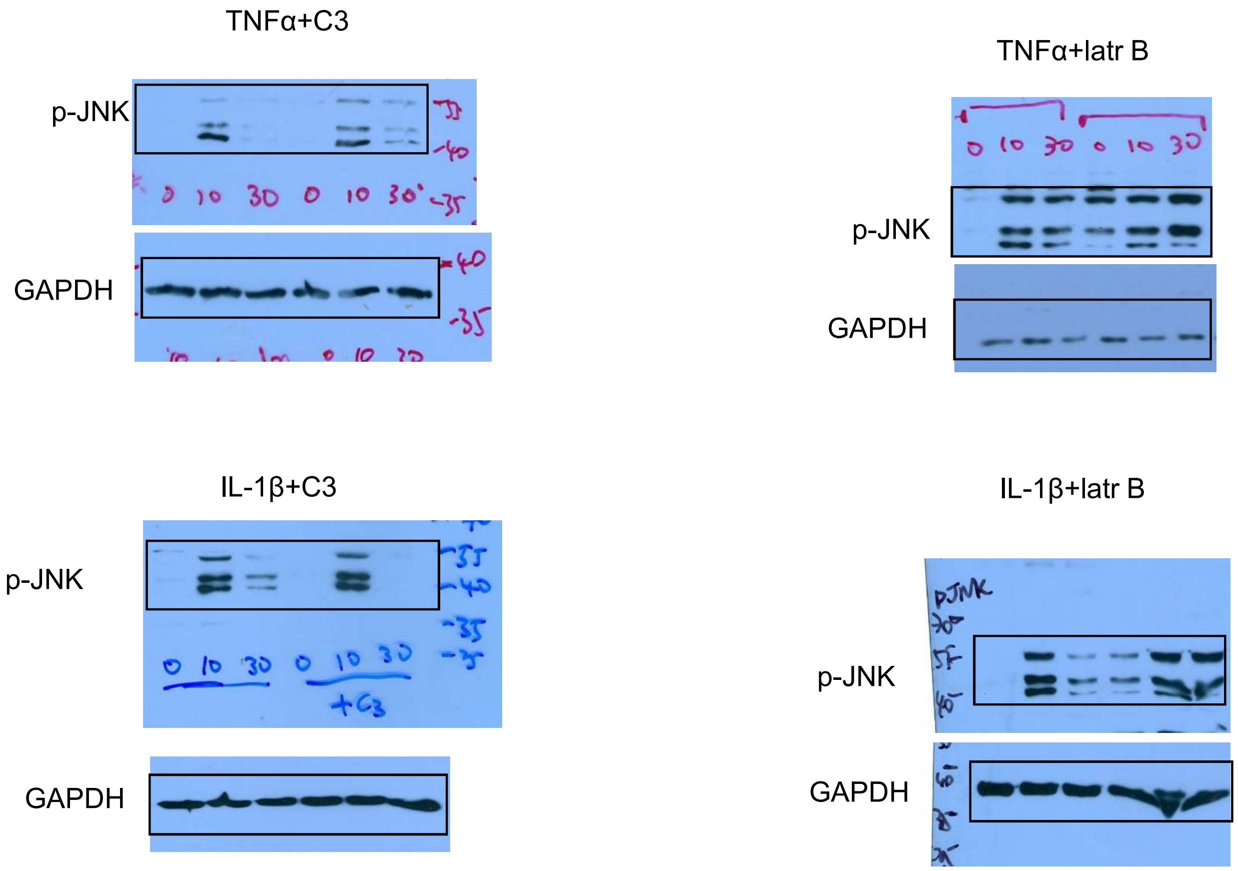

Supplement: Supplementary file 1 — Supplementary Information [file 41467_2020_19229_MOESM1_ESM.pdf]
